# Saddlepoint approximations for linear rank tests with left-truncated, right-censored, and cross-sectional data under randomized block design

**DOI:** 10.1038/s41598-026-59404-y

**Published:** 2026-06-30

**Authors:** Kholoud S. Kamal, Abd El-Raheem M. Abd El-Raheem, Mahmoud M. Ramadan

**Affiliations:** Department of Mathematics, Faculty of Education, Ain Shams University, Cairo, Egypt

**Keywords:** Truncation, Censoring, Cross-sectional study, Linear rank tests, Randomized block design, Mid *p* value, Saddlepoint approximation, Engineering, Mathematics and computing, Medical research

## Abstract

Left-truncated data arise when events are only recorded if they occur after a pre-specified time point, while right censoring occurs when the exact event time is not fully observed. Cross-sectional data refer to data collected at a single time point without follow-up. This paper proposes saddlepoint approximations (SPA) for the mid *p* values of four linear rank test statistics, namely $T^{b}_{LR}$, $T^{b}_{WLR}$, $T^{b}_{LRC}$, and $T^{b}_{WC}$, applied to these data types under a randomized block design (RBD). These test statistics are newly adapted to the RBD framework, and the Skovgaard SPA formula is applied to derive accurate approximations for their mid *p* values. The accuracy of the proposed SPA is compared against the standard normal approximation (NA) via extensive simulation studies under extreme value and logistic distributions, and illustrated through real data examples. Results demonstrate that the SPA consistently provides more accurate approximations to the mid *p* values compared to the NA across all simulation scenarios and real data examples.

## Introduction

Time-to-event data arise naturally in biomedical, epidemiological, and clinical investigations where the primary objective is to evaluate the latency period preceding a specific event, such as mortality, disease recurrence, or therapeutic failure. In practice, survival datasets are frequently incomplete due to constraints in observational designs, delayed subject entry, loss to follow-up, or truncated monitoring windows. Such observational complications introduce substantial analytical challenges, particularly when the sample fails to fully represent the underlying target population. Consequently, specialized statistical frameworks are indispensable to adjust for these partial observations and to guarantee valid asymptotic or exact inference. The two most prevalent mechanisms resulting in incomplete survival observations are truncation and censoring. Truncation occurs when individuals are sampled conditionally upon their event times falling within a specific observable region. Let *Y* denote the event time of interest. Under double truncation, the event is observed if and only if $Y \in (L_t, U_t)$, where $L_t$ and $U_t$ represent the lower and upper truncation boundaries, respectively. Shen^1^ provides a comprehensive discussion of this phenomenon and its implications for statistical analysis. In many practical applications, however, the truncation mechanism is one-sided. Right truncation limits the sample to subjects whose event occurs before a specific boundary $U_t$. As a result, events occurring after this point are systematically excluded from the study, as noted by Chi et al.^2^. Conversely, left truncation restricts the analysis exclusively to individuals satisfying $Y > L_t$, a scenario frequently encountered in cohorts where subjects only become eligible for study enrollment after an initiating event has already transpired. For further details, see Klein and Moeschberge^3^, Ying^4^, Hosmer et al.^5^, and Cohen^6^. Censoring constitutes another fundamental characteristic of survival analysis, occurring when the exact event time cannot be precisely observed, as noted by Klein and Moeschberger^3^. Among the various censoring mechanisms, right censoring is the most prevalent, arising when the event of interest is unobserved prior to study termination or when a subject is lost to follow-up. In contrast, left censoring occurs when the event is known to have taken place prior to a specific baseline time point, as noted by Dey et al. ^7^, whereas interval censoring signifies that the exact event time is bounded within a known, finite time interval, as described by Shen^8^.

In many biomedical studies, left truncation and right censoring occur together, which complicates the statistical inference. This joint data structure commonly arises in cohort follow-up studies, where subjects who have experienced an initiating event are monitored until a terminal event occurs or the study ends. Subjects whose event times are not captured within this period are treated as right-censored. The problem of two-sample testing under both left truncation and right censoring has been addressed by several authors, including Ning et al.^9^, Lagakos et al.^10^, Chan and Qin^11^, and Chi et al.^2^, who proposed different methods to handle these joint complexities.

A related framework involves length-biased right-censored data, where subjects with longer survival times are more likely to be included in the sample. This occurs when sampling is conducted over a fixed time interval, making the truncation times uniformly distributed across the sampling window Qin et al.^12^. Consequently, the observed sample is no longer representative of the target population, and standard statistical procedures will yield biased inferences if this length-bias effect is ignored.

Another important setting involves cross-sectional survival data collected without longitudinal follow-up. In this case, observations are recorded at a single sampling point, capturing only the backward recurrence time–the duration between the initiating event and the sampling time. Because individuals who experience the event before the sampling point are systematically excluded, these data are inherently left-truncated. As noted by Chan and Qin^11^, this selection mechanism introduces a clear bias that must be accounted for, rendering standard survival analysis methods directly inapplicable.

In many clinical studies involving survival outcomes, these complex data structures are analyzed within randomized experimental designs. Randomization plays a fundamental role in clinical trial design by reducing selection bias and ensuring comparability between treatment groups. Common randomization designs include complete randomization, random allocation, and randomized block designs. When data are collected across multiple clinical studies, and the goal is to minimize selection bias and control for sample size imbalance, the randomized block design is often the most appropriate choice (see Matts and Lachin^13^, Lachin et al.^14^, and Efird^15^). In this design, the total sample of *N* patients is partitioned into *B* blocks of equal size, typically 2*n*. Within each block, *n* patients are randomly assigned to the treatment group *T* and *n* to the control group *C*, ensuring that all $$\left( {\begin{array}{c}2n\\ n\end{array}}\right)$$ randomizations of the *T*’s and the *C*’s labels are uniformly distributed. This approach was originally proposed by Zelen^16^ and Matthews^17^. A more flexible extension of the randomized block design is the generalized randomized block design, which accommodates varying block sizes and allows for unequal group allocations within each block. Suppose a total of *N* patients are divided into *B* blocks, where block *i* contains $n_{i}$ patients such that $N=\sum \nolimits _{i=1}^{B}n_{i}$. Within each block, a random allocation rule is applied to assign $m_{i}$ patients to group *T* and the remaining $n_{i}-m_{i}$ patients to group *C*.

Statistical inference under such randomization structures is often based on linear rank test procedures. Linear rank tests are widely used in survival analysis because of their robustness and minimal distributional assumptions. However, constructing accurate test procedures becomes substantially more challenging in the presence of left truncation, right censoring, and block randomization. Exact permutation-based inference may become computationally intensive, particularly when the number of possible treatment allocations is large. Moreover, standard asymptotic approximations based on the normal distribution may perform poorly in small or moderate sample settings, especially when accurate tail probability estimation is required. These limitations motivate the development of more accurate approximation techniques for hypothesis testing in complex survival data structures.

One particularly effective approach for improving small-sample inference is the SPA. This higher-order method is especially critical in randomized survival studies with limited sample sizes, where standard first-order asymptotic methods, specifically normal approximation, frequently yield substantial errors. In many practical applications, the primary inferential focus centers on tail probabilities and *p* values, which are highly sensitive to the accuracy of the underlying approximation. While exact permutation methods provide reliable results, they remain computationally demanding and difficult to implement under complex censoring and truncation mechanisms. Consequently, saddlepoint techniques offer a compelling alternative by retaining extreme accuracy while remaining computationally efficient. SPA methods provide an attractive solution to these challenges. SPA methods are well known for providing highly accurate approximations to probability density functions, cumulative distribution functions, and tail probabilities, particularly in small-sample settings. Unlike classical asymptotic approximations derived from the Central Limit Theorem, which typically exhibit an error of order $O(N^{-1/2})$, SPA methods generally achieve a higher-order accuracy of order $O(N^{-3/2})$. These methods utilize the cumulant generating function or moment generating function of the underlying statistic and are especially effective when accurate tail behavior is required.

Foundational developments include the seminal work of Daniels^18^ on density approximations, followed by extensions to cumulative distribution functions and multivariate settings by Lugannani and Rice^19^, Skovgaard^20^, and Wang^21^. Following these foundational developments, SPA methods have been successfully applied across numerous areas of statistical inference, encompassing hypothesis testing, permutation procedures, and linear rank statistics. Crucial advancements were established by Daniels^22^, Davison and Hinkley^23^, Butler^24^, and Abd-Elfattah and Butler^25,26^, among others. More recently, SPA techniques have been extended to rank-based procedures under randomized experimental designs, including generalized randomized block designs and related allocation schemes Abd-Elfattah^27^, Kamal and Abd El-Raheem^28^. In parallel, modern statistical methodology has increasingly focused on developing precise inferential tools for censored and heterogeneous data structures, such as censored panel data models and latent group frameworks Ando and Seo^29^ Dai et al.^30^ Tong et al.^31^. These concurrent developments emphasize the ongoing necessity for accurate and computationally efficient approximation methods within complex survival frameworks.

Despite these important advances, several inferential challenges remain unresolved. Although substantial progress has been made in the development of SPAs and rank-based inference procedures, relatively limited attention has been devoted to survival settings involving left truncation, right censoring, and cross-sectional sampling under RBD. In particular, the accurate approximation of exact mid *p* values for linear rank tests in these settings remains computationally challenging, especially in small-sample situations where standard normal approximations may be unreliable. This gap motivates the present study. The present paper aims to address these challenges through the development and evaluation of SPA-based inferential procedures.

This paper develops SPA technique for linear rank tests under left-truncated right-censored data, as well as cross-sectional survival data within RBD. The proposed method is applied to approximate exact mid *p* values, allowing for a direct comparison with the NA regarding inferential accuracy. We assess the performance of this proposed procedure through comprehensive simulation studies and illustrate its utility using real data applications. Ultimately, this SPA yields highly accurate approximations and substantially outperforms the conventional normal approximation, especially in small-sample settings.

The remainder of this paper is organized as follows. In “Applying linear rank tests for survival-type data with left truncation, right censoring and cross-sectional data under randomized block design” section introduces linear rank test procedures tailored to settings involving left-truncated, right-censored data and cross-sectional data without follow-up under a randomized block design. In “Double SPA method” section presents the technical framework of the double SPA method, while “Simulation results” section illustrates the practical relevance of the proposed techniques through the analysis of empirical datasets. In “Data case studies” section evaluates the accuracy of the SPA method in approximating EMPVs through a series of controlled simulation experiments. Finally, “Concluding remarks and future works” section summarizes the primary findings and outlines potential directions for further research.

## Applying linear rank tests for survival-type data with left truncation, right censoring and cross-sectional data under randomized block design

This section examines nonparametric testing approaches for datasets characterized by left truncation and right censoring, as well as for cross-sectional data without follow-up, all within the context of a randomized block design. Linear rank tests are widely used in two-sample comparisons between independent groups to assess the impact of a new treatment versus a standard one.

Within the randomized block design framework, consider a sample of *N* patients divided into *B* blocks, where block *i* contains $n_{i}$ patients such that $N=\sum \nolimits _{i=1}^{B}n_{i}$, $\forall i=1,2,\ldots ,B$. Within each block *i*, the random allocation procedure assigns $m_{i}$ patients to group *T* and the remaining $n_{i}-m_{i}$ patients to group *C*, $\forall i=1,2,\ldots ,B$. The statistical methodology of Ning et al.^9^ is extended here to the randomized block design setting. Let $\{(a_{ij},y_{ij},z_{ij},\delta _{ij});\ i=1,\ldots ,B,\ j=1,\ldots ,n_{i}\}$ denote the observed sample, subject to $a_{ij} \le y_{ij}$ for all *i* and *j*. Here, $a_{ij}$ is the left truncation time for the $j^{th}$ patient in block *i*, representing the time between initial diagnosis and study enrollment; $y_{ij}$ is the observed time, corresponding to either failure or censoring; $z_{ij}$ is the group indicator, with $z_{ij}=1$ for group *T* and $z_{ij}=0$ for group *C*; and $\delta _{ij}$ is the censoring indicator, with $\delta _{ij}=1$ if the event occurred and $\delta _{ij}=0$ if the observation was censored. Let $W_{T}(t)$ and $W_{C}(t)$ denote the survival functions for groups *T* and *C*, respectively, for $t\ge 0$. The log-rank test evaluates the null hypothesis $H_{0}: W_{T}(t) = W_{C}(t)$ for all $t\ge 0$ against either a one-sided or two-sided alternative. Within block *i*, let $t_{i(1)}< t_{i(2)}< \cdots < t_{i(h_{i})}$ denote the distinct ordered failure times, where $h_{i}$ is the number of such times in block *i*. Let *h* denote the total number of distinct failure times across the full dataset. Ning et al.^9^ proposed the log-rank statistic

1 $$\begin{aligned} T_{LR}=\sum _{\nu =1}^{h}\left( O_{2\nu }-N_{2\nu } \frac{O_{\nu }}{N_{\nu }}\right) , \end{aligned}$$

where, at each failure time $t_{\nu }$: $O_{1\nu }$, $O_{2\nu }$, and $O_{\nu }=O_{1\nu }+O_{2\nu }$ denote the observed failures in groups *T*, *C*, and the combined sample, respectively; and $N_{1\nu }$, $N_{2\nu }$, and $N_{\nu }=N_{1\nu }+N_{2\nu }$ denote the corresponding numbers at risk, for $\nu =1,\ldots ,h$. Ning et al.^9^ established the robustness of this statistic under left truncation, assuming equivalent censoring mechanisms across groups.

Following the approach of Van Elteren^32^, we assign the classical Van Elteren weights, $b_{i}=1/(n_{i}+1)$, to each block *i*. These weights are optimal because they minimize the variance of the combined test statistic under the null hypothesis, thereby maximizing the test’s statistical power. The statistic in Eq. (1) is then reformulated as

2 $$\begin{aligned} T^{b}_{LR}=\sum _{i=1}^{B} b_{i} \sum _{j=1}^{h_{i}} \left( O_{i2j}-N_{i2j} \frac{O_{ij}}{N_{ij}}\right) , \end{aligned}$$

where, at failure time $t_{i(j)}$ within block *i*: $O_{i1j}$, $O_{i2j}$, and $O_{ij}=O_{i1j}+O_{i2j}$ are the observed failures in groups *T*, *C*, and the combined sample; and $N_{i1j}$, $N_{i2j}$, and $N_{ij}=N_{i1j}+N_{i2j}$ are the corresponding numbers at risk, for $i=1,\ldots ,B$ and $j=1,\ldots ,h_{i}$. The statistic $T^{b}_{LR}$ may be written to show individual-level contributions within each block as

3 $$\begin{aligned} T^{b}_{LR}=\sum _{i=1}^{B} b_{i} \sum _{k=1}^{n_{i}} z_{ik} \sum _{j=1}^{h_{i}}\left( O^{*}_{ikj}-N^{*}_{ikj} \frac{O_{ij}}{N_{ij}}\right) , \end{aligned}$$

where $O^{*}_{ikj}=I(y_{ik} = t_{i(j)})\,\delta _{ik}$ indicates whether patient *k* in block *i* failed at time $t_{i(j)}$, and $N^{*}_{ikj}=I(y_{ik} \ge t_{i(j)})$ indicates whether that patient was at risk at that time.

The reverse-time weighted log-rank test, introduced by Lagakos et al.^10^, was originally designed for right-truncated data, where only patients whose failure times fall below a specified threshold are observed. The method reverses the time scale and applies a weighted log-rank test to the transformed data. Chi et al.^2^ reviewed this approach, noting its practical utility while observing that its weight function, which depends on cumulative hazard functions, may be difficult to interpret. The test statistic is adapted here to accommodate the randomized block design.

The right-truncated dataset is defined as $\{(x_{ik}, b_{ik}, z_{ik});\ i=1,\ldots ,B,\ k=1,\ldots ,n_{i}\}$, where $x_{ik} \le b_{ik}$ for all *i* and *k*. Here, $x_{ik}$ is the observed survival time, $b_{ik}$ is the truncation time, and $z_{ik}$ is the group indicator for patient *k* in block *i*, with $z_{ik}=1$ for group *T* and $z_{ik}=0$ for group *C*.

Let $x_{i(1)}< x_{i(2)}< \cdots < x_{i(m_{i})}$ denote the distinct ordered failure times in block *i*, where $m_{i}$ is their total number. Following Lagakos et al.^10^, survival times are reversed via $R = \tau - x$, where $\tau$ is the fixed study end date, common to all patients, chosen such that $\tau \ge \max _{i,k}\{x_{ik}\}$ to ensure all reversed times are non-negative. Since $x \le b$ implies $\tau - x \ge \tau - b$, this transformation converts right-truncated data into a left-truncated format, with $\tau - b$ as the new truncation time. The distinct ordered reversed survival times in block *i* are denoted $r_{i(1)}< r_{i(2)}< \cdots < r_{i(m_{i})}$. Under this transformation, patient *k* in block *i* is at risk at reversed time $r_{i(d)}$ if and only if $\tau - x_{ik} \ge r_{i(d)} \ge \tau - b_{ik}$, meaning the patient had not yet failed and had already entered the study on the original time scale.

The null hypothesis is $H_{0}: S_{1}^{*}(x) = S_{2}^{*}(x)$ over $(0, \tau )$, where $S_{1}^{*}(x)$ and $S_{2}^{*}(x)$ denote the conditional survival functions for groups *T* and *C*, respectively. The test is conducted against either a one-sided or two-sided alternative.

Let *m* denote the total number of distinct failure times in the observed sample, $\psi _{uw}$ the number of failures in group *u* at reversed time $r_{(w)}$, and $\pi _{uw}$ the corresponding number at risk, for $u=1,2$ and $w=1,\ldots ,m$. The weight function is $\xi _{w} = (\tau - r_{(w)})^{\gamma }$, where $\gamma > 0$ is a positive constant. The reverse-time weighted log-rank statistic is then

4 $$\begin{aligned} T_{WLR}=\sum _{w=1}^{m}\xi _{w}\left( \psi _{2w}-\pi _{2w} \frac{\psi _{w}}{\pi _{w}}\right) , \end{aligned}$$

where $$\psi _{w} = \psi _{1w} + \psi _{2w}$$ and $$\pi _{w} = \pi _{1w} + \pi _{2w}$$.

Under the randomized block design, let $\xi _{id} = (\tau - r_{i(d)})^{\gamma }$ denote the weight for the $d^{th}$ reversed failure time in block *i*, and let $\psi _{iud}$ and $\pi _{iud}$ denote the number of failures and patients at risk in group *u* at reversed time $r_{i(d)}$, for $u=1,2$, $i=1,\ldots ,B$, and $d=1,...,m_{i}$. The statistic $T_{WLR}$ is reformulated as

5 $$\begin{aligned} T^{b}_{WLR}=\sum _{i=1}^{B} b_{i} \sum _{d=1}^{m_{i}} \xi _{id}\left( \psi _{i2d}-\pi _{i2d} \frac{\psi _{id}}{\pi _{id}}\right) , \end{aligned}$$

where $$\psi _{id} = \psi _{i1d} + \psi _{i2d}$$ and $$\pi _{id} = \pi _{i1d} + \pi _{i2d}$$.

The statistic $T^{b}_{WLR}$ may be written to show individual-level contributions within each block as

6 $$\begin{aligned} T^{b}_{WLR}=\sum _{i=1}^{B} b_{i} \sum _{k=1}^{n_{i}} z_{ik} \sum _{d=1}^{m_{i}}\xi _{id}\left( \psi ^{*}_{ikd}-\pi ^{*}_{ikd} \frac{\psi _{id}}{\pi _{id}}\right) , \end{aligned}$$

where $\psi ^{*}_{ikd} = I(\tau - x_{ik} = r_{i(d)})$ indicates whether patient *k* in block *i* failed at reversed time $r_{i(d)}$, and $\pi ^{*}_{ikd} = I(\tau - x_{ik} \ge r_{i(d)} \ge \tau - b_{ik})$ indicates whether that patient was at risk at that time.

Following the previous discussion of test statistics under randomized block design, we introduce two additional statistics based on cross-sectional data, which do not involve patient follow-up Chan and Qin^11^. This type of data captures information at a single point in time, focusing on patients who have already experienced the initial event, such as a disease diagnosis. In this context, only the backward recurrence time, defined as the duration from the initial event to the moment of sampling, is observed. In other words, the backward recurrence time quantifies how long the patient has survived since the initial event. A key feature of such data is left truncation, as patients who experienced the failure event (e.g., death from the disease) before the study’s sampling time are systematically excluded. In cross-sectional studies lacking follow-up under randomized block design, the only observable quantity is the backward recurrence time in each block *i*, denoted by $\upsilon _{i}$. This variable captures the interval between the initial event, such as disease diagnosis, and the time of recruitment. The observed recurrence times within block *i* are ordered as $\upsilon _{i(1)}<\upsilon _{i(2)}< \cdots <\upsilon _{i(n_{i})}$, with the corresponding group indicators $z_{i(1)},z_{i(2)},\ldots ,z_{i(n_{i})}$. For the purposes of statistical inference, the backward recurrence times within block *i* are denoted as $\upsilon _{i1},\ldots ,\upsilon _{in_{i}}$, are analyzed as if they represent complete survival times drawn from an unbiased sample. This assumption permits the use of conventional survival analysis techniques, even in the absence of longitudinal follow-up data. In the context of cross-sectional survival data without longitudinal follow-up, the null hypothesis is formulated as $H_{0}: G_{T}(\upsilon )=G_{C}(\upsilon )$, $\forall \upsilon >0$, where $G_{T}(\upsilon )$ and $G_{C}(\upsilon )$ represent the survival functions corresponding to groups *T* and *C*, respectively, based on the observed backward recurrence times. Let $z_{(g)}$ denote the group indicator for the $g^{th}$ ordered observation, and let *n* denote the total sample size within the block. Following Chan and Qin^11^, the log-rank test statistic for cross-sectional survival data without follow-up is expressed as:

7 $$\begin{aligned} T_{LRC}=\sum _{g=1}^{n} z_{(g)} (n^{-1}+(n-1)^{-1}+ \cdots +(n+g-1)^{-1} -1), \end{aligned}$$

The statistic $T_{LRC}$ may be rewritten under the randomized block design as:

8 $$\begin{aligned} T^{b}_{LRC}=\sum _{i=1}^{B} b_{i} \sum _{k=1}^{n_{i}} z_{i(k)} (n_{i}^{-1}+(n_{i}-1)^{-1}+ \cdots +(n_{i}+k-1)^{-1} -1), \end{aligned}$$

The Wilcoxon test statistic is given by the following expression:

9 $$\begin{aligned} T_{WC}=\sum _{g=1}^{n} z_{(g)} (2g/(n+1)-1), \end{aligned}$$

The preceding statistic is rewritten for individual-level block contributions:

10 $$\begin{aligned} T^{b}_{WC}=\sum _{i=1}^{B} b_{i} \sum _{k=1}^{n_{i}} z_{i(k)} (2k/(n_{i}+1)-1). \end{aligned}$$

The four statistics $T^{b}_{LR}$, $T^{b}_{WLR}$, $T^{b}_{LRC}$, and $T^{b}_{WC}$ share a common structure. Specifically, defining $q_{ik}$ as the score assigned to the $k^{th}$ patient in block *i*, where $q_{ik}$ takes a distinct form for each statistic, all four may be written in the unified form:

11 $$\begin{aligned} T^{b}_{stat.}=\sum _{i=1}^{B} b_{i} \sum _{k=1}^{n_{i}} z_{ik} q_{ik}, \end{aligned}$$

where $m_{i}$ denotes the number of patients assigned to group *T* within block *i*. Under the random allocation design, Abd-Elfattah^27^ established that $\mu _{T^{b}_{stat.}}=E(T^{b}_{stat.})=0$ and:

12 $$\begin{aligned} \sigma ^{2}_{T^{b}_{stat.}}=V(T^{b}_{stat.}) = \sum _{i=1}^{B} \frac{m_{i}(n_{i}-m_{i})}{n_{i}(n_{i}-1)} b^{2}_{i} \sum _{k=1}^{n_{i}} q^{2}_{ik}, \end{aligned}$$

It is worth noting the behavior of the proposed statistics under simplified settings. In the special case of a single block ($B=1$), the block weight $b_1$ factors out as a positive constant, causing all four combined statistics to reduce directly to their single-sample counterparts. Additionally, in the absence of left truncation and right censoring, the score functions simplify to their standard configurations, meaning our unified statistics reduce exactly to the classical log-rank and Wilcoxon forms under randomized designs.

## Double SPA method

We apply the SPA method to demonstrate its accuracy and computational efficiency in approximating the EMPVs of the linear rank statistic $T^{b}_{stat.}$, as defined in Eq. 11. For computational convenience, $T^{b}_{stat.}$ may be equivalently expressed as:

13 $$\begin{aligned} T^{b}_{stat.}=\sum _{i=1}^{B}\sum _{k=1}^{n_{i}} z_{ik} bq_{ik}, \end{aligned}$$

where $bq_{ik}=b_{i}q_{ik}$. The random vector associated with block *i*; $(z_{i1},z_{i2},\ldots ,z_{in_{i}})$ where $i=1,\ldots ,B$, is assumed to follow a uniform distribution based on the random allocation design utilized within block *i*. Specifically, the allocation is equally likely over all $$\left( {\begin{array}{c}n_{i}\\ m_{i}\end{array}}\right) ^{-1}$$ possible arrangements, with $m_i$ patients assigned to group *T* and $(n_{i} - m_{i})$ patients assigned to group *C*. Each block’s allocation vector is generated independently, so $(z_{i1}, \ldots , z_{in_{i}})$ is independent of $(z_{r1}, \ldots , z_{rn_r})$ for any $r \ne i$.

The statistic $T^{b}_{stat.}$ as given in Eq. 13 depends on the treatment allocation indicators $z_{ik}$, where $i = 1, \ldots , B$ and $k = 1, \ldots , n_{i}$. Let $\zeta _{i1}, \zeta _{i2}, \ldots , \zeta _{in_{i}}$ be independent and identically distributed (i.i.d.) Bernoulli($\xi _{i}$) random variables for each $i = 1, \ldots , B$, independent across different blocks, where $\mid$ denotes conditioning on the fixed treatment totals within each block. Under the randomization mechanism, the joint distribution of $(z_{11}, z_{12}, \ldots , z_{Bn_{B}})$ is equivalent to the conditional distribution:

14 $$\begin{aligned} \begin{aligned} z_{11}, z_{12}, \ldots , z_{Bn_{B}}&\overset{D}{\sim } \zeta _{11}, \zeta _{12}, \ldots , \zeta _{Bn_{B}} \\&\;\Big |\; \sum _{k=1}^{n_{1}} \zeta _{1k} = m_{1},\; \ldots ,\; \sum _{k=1}^{n_{B}} \zeta _{Bk} = m_{B}. \end{aligned} \end{aligned}$$

This equivalence holds because, under the random allocation design, each arrangement of $m_{i}$ ones and $(n_{i}-m_{i})$ zeros within block *i* is equally likely. Conditioning independent Bernoulli random variables $\zeta _{ik}$ on their sum equaling $m_{i}$ produces exactly this uniform distribution over all $$\left( {\begin{array}{c}n_{i}\\ m_{i}\end{array}}\right)$$ possible arrangements. This conditional Bernoulli representation is the key step that enables the application of the saddlepoint approximation, as it provides a tractable cumulant generating function (CGF) for the test statistic $T^{b}_{stat.}$. Accordingly, the statistic $T^{b}_{\text {stat.}}$ as presented in Eq. 13 can be represented as

15 $$\begin{aligned} \begin{aligned} T^{b}_{\text {stat.}}&= \sum _{i=1}^{B} \sum _{k=1}^{n_{i}} z_{ik} bq_{ik} \overset{D}{\sim }\ \sum _{i=1}^{B} \sum _{k=1}^{n_{i}} \zeta _{ik} bq_{ik} \\&\;\Big |\; \sum _{k=1}^{n_{1}} \zeta _{1k} = m_{1},\; \sum _{k=1}^{n_{2}} \zeta _{2k} = m_{2},\; \ldots ,\; \sum _{k=1}^{n_{B}} \zeta _{Bk} = m_{B}. \end{aligned} \end{aligned}$$

Let $T^{b}_{0}$ denote the observed value of the statistic $T^{b}_{stat.}$ as formulated in Eq. 13. The EMPV corresponding to $T^{b}_{stat.}$ at $T^{b}_{0}$ is defined as $mid\text {-}pv(T^{b}_{0}) = Pr(T^{b}_{stat.} >T^{b}_{0}) + \tfrac{1}{2} Pr(T^{b}_{stat.} = T^{b}_{0})$.

To approximate this EMPV, the double SPA method is employed for the conditional tail probability $$Pr(\sum {\sum {\zeta _{{ik}} } } bq_{{ik}} \ge T_{0}^{b} {\mid }$$$$\sum {\zeta _{{1k}} } = m_{1} , \ldots ,\sum {\zeta _{{Bk}} } = m_{B} )$$. According to Skovgaard^20^, let $$M=(m_{1},m_{2},\ldots ,m_{B})$$ denote the vector of treatment group sizes across blocks, and let $$(\hat{s}, \hat{L})=(\hat{s},\hat{l}_{1},\hat{l}_{2},\ldots ,\hat{l}_{B})$$ and $$\hat{L}_{0}=(\hat{l}_{10},\hat{l}_{20},\ldots ,\hat{l}_{B0})$$ denote the numerator and denominator saddlepoints, respectively. The joint CGF, *K*(*s*, *L*), corresponding to the random vector $$\big(\sum\limits_{{i = 1}}^{B} {\sum\limits_{{k = 1}}^{{n_{i} }} {\zeta _{{ik}} } } bq_{{ik}}$$, $$\sum\limits_{{k = 1}}^{{n_{i} }} {\zeta _{{1k}} } , \ldots ,\sum\limits_{{k = 1}}^{{n_{B} }} {\zeta _{{Bk}} } \big)$$, is given by

$$\begin{aligned} K(s,L)=\sum _{i=1}^{B}\sum _{k=1}^{n_{i}}\ln \{(1-\xi _{i})+ \xi _{i}\exp (bq_{ik}s+l_{i})\}. \end{aligned}$$

Here, $K^{\prime \prime }(\hat{s},\hat{L})$ denotes the $(B+1)\times (B+1)$ Hessian matrix of *K*(*s*, *L*) evaluated at $(\hat{s},\hat{L})$, and $K^{\prime \prime }_{LL}(0,\hat{L}_{0})$ denotes the $B\times B$ sub-block $\partial ^2/\partial L\partial L^{T}$ of $K^{\prime \prime }(\cdot )$ evaluated at $(0,\hat{L}_{0})$. The numerator saddlepoint $(\hat{s},\hat{l}_{1},\hat{l}_{2},\ldots , \hat{l}_{B})$ solves the saddlepoint equations $K^{\prime }(\hat{s},\hat{L})=(T^{b}_{0},m_{1},m_{2},\ldots ,m_{B})$:

$$\begin{aligned} K^{\prime }_{s}(\hat{s},\hat{L})=\sum _{i=1}^{B}\sum _{k=1}^{n_{i}} \left( \frac{bq_{ik}\xi _{i}\exp (bq_{ik}\hat{s}+\hat{l}_{i})}{(1-\xi _{i})+\xi _{i}\exp (bq_{ik}\hat{s}+\hat{l}_{i})}\right) =T^{b}_{0}, \end{aligned}$$

$$\begin{aligned} K^{\prime }_{l_{i}}(\hat{s},\hat{L})=\sum _{k=1}^{n_{i}} \left( \frac{\xi _{i}\exp (bq_{ik}\hat{s}+\hat{l}_{i})}{(1-\xi _{i})+\xi _{i}\exp (bq_{ik}\hat{s}+\hat{l}_{i})}\right) =m_{i}, \quad i=1,\ldots ,B. \end{aligned}$$

The denominator saddlepoint $\hat{L}_{0} = (\hat{l}_{10}, \hat{l}_{20}, \ldots , \hat{l}_{B0})$ is determined by solving

$$\begin{aligned} K^{\prime }_{l_{i}}(0, \hat{L}_{0}) = \sum _{k=1}^{n_{i}}\left( \frac{\xi _{i}\exp (\hat{l}_{i0})}{(1-\xi _{i})+\xi _{i}\exp (\hat{l}_{i0})} \right) = m_{i}, \quad i = 1, \ldots , B. \end{aligned}$$

Because the randomization distribution does not depend on $\xi _{i}$, assigning $\xi _{i} = \frac{m_{i}}{n_{i}}$, $\forall$ $i = 1, \ldots , B$, yields the explicit denominator saddlepoint solution $\hat{l}_{i0} = 0$, $i = 1, \ldots , B$, thereby simplifying the computational procedure. The symbols $\phi$ and $\Phi$ denote the probability density function and cumulative distribution function of the standard normal distribution, respectively. The quantities $\hat{\eta _{1}}$ and $\hat{\eta _{2}}$ are defined as:

$$\begin{aligned} \hat{\eta _{1}}=sgn(\hat{s})[2\{(K(0,\hat{L}_{0})-M^{T}\hat{L}_{0})- (K(\hat{s},\hat{L})-M^{T}\hat{L}-T^{b}_{0}\hat{s})\}]^{1/2}, \end{aligned}$$

$$\begin{aligned} \hat{\eta _{2}}=\hat{s}\left[ {\frac{\mid K^{\prime \prime }(\hat{s}, \hat{L})\mid }{\mid K^{\prime \prime }_{LL}(0,\hat{L}_{0})\mid }} \right] ^{1/2}. \end{aligned}$$

The double SPA to the EMPV is then given by

16 $$\begin{aligned} mid\text {-}pv(T^{b}_{0}) \simeq 1 - \Phi (\hat{\eta _{1}}) - \phi (\hat{\eta _{1}}) \left( \frac{1}{\hat{\eta _{1}}} - \frac{1}{\hat{\eta _{2}}} \right) . \end{aligned}$$

The continuous SPA form in Eq. 16 is employed because the test statistic $T^{b}_{\text {stat.}}$ is non-lattice, meaning that its values are not equally spaced and do not fall on a regular discrete grid. Although the underlying permutation distribution is technically discrete, its support becomes highly irregular due to the combination of the block weights ($b_i$) and the fractional score functions ($q_{ik}$). Consequently, standard continuity corrections cannot be applied, and the continuous approximation yields a smoother, more reliable approximation of the mid *p*-value than discrete alternatives, as theoretically justified by Butler^24^ and Davison and Wang^33^. This approach is strongly validated by our simulation results across all evaluated sample sizes ($N = 15, 20, 40, 60, 80$). Even at the smallest nominal size ($N = 15$), where the discrete nature of the permutations is most pronounced, the stable Type-I error rates and low relative errors confirm that any approximation error due to ignoring discreteness is entirely negligible.

## Simulation results

A comprehensive simulation study is undertaken to evaluate both the accuracy and computational efficiency of the SPA method across a spectrum of sample sizes and distributional scenarios within the framework of linear rank statistics. The accelerated failure time (AFT) model is applied to censored survival data, modeling the logarithm of survival time as a function of explanatory covariates. This modeling paradigm effectively characterizes the manner in which covariates influence the acceleration or deceleration of the event time, thereby quantifying their impact on the timing of the event of interest. Let *y* denote the failure time and *z* represent a vector of covariates, potentially time-dependent. When all covariates are time-independent, the AFT model assumes the following log-linear representation:

17 $$\begin{aligned} \log (y) = \beta z + \epsilon , \end{aligned}$$

where $\epsilon$ is a random error term independent of *z*, and $\beta$ denotes the corresponding regression coefficient. In a two-sample comparison, the covariate *z* indicates the treatment group for each subject.

### Procedure for simulation data generation

Data generation under the AFT model proceeded as follows:

1. Failure times *y* were generated from a weibull(2.5, 50), exponential(0.02), log-logistic(0, 1), logistic(55, 2) or extreme value(0, 0, 1) distribution.
2. Truncation variables *u* were generated from a uniform distribution truncated to the interval (20, 100).
3. Data generation is conducted across five sample size configurations: $N = 15, 20, 40, 60,$ and 80. For the smallest cohort ($N = 15$), a generalized randomized block design is implemented utilizing $B = 4$ blocks of sizes $n_1 = 4, n_2 = 4, n_3 = 4,$ and $n_4 = 3$, where the corresponding number of patients allocated to the treatment group within each block is $m_1 = 2, m_2 = 2, m_3 = 2,$ and $m_4 = 1$, respectively. For the remaining configurations ($N = 20, 40, 60,$ and 80), a standard balanced randomized block design is employed. Specifically, $B = 5$ blocks of equal size are used for $N = 20$ and $N = 40$, whereas $B = 10$ blocks of equal size are utilized for $N = 60$ and $N = 80$. Within each of these balanced configurations, patients in every block are equally allocated between the treatment and control groups.
4. *N* patients were divided into *B* blocks, each containing $n_{i}$ patients. Within each block, $m_{i}$ were randomly assigned to group *T* with treatment indicator $z=1$, and the remainder to group *C* with treatment indicator $z=0$.
5. The observed value of the test statistic was computed:
  $$T^{b}_{\text {stat}} = \sum _{i=1}^{B} b_i \sum _{k=1}^{n_i} z_{ik} q_{ik},$$
  along with its mean and variance:
  $$\mu _{T^{b}_{stat.}} = E(T^{b}_{\text {stat}}) = 0,$$
  $$\sigma ^2_{T^{b}_{stat.}} = V(T^{b}_{stat.}) = \sum _{i=1}^{B} \frac{m_i (n_i - m_i)}{n_i (n_i - 1)} b_i^2 \sum _{k=1}^{n_i} q_{ik}^2.$$
6. The mid *p* value of $T^{b}_{stat.}$ at the observed value $T^{b}_{0}$, referred to as the NA *p* value, is approximated using the normal approximation:
  $$\Pr (T^{b}_{stat.} \ge T^{b}_{0}) \simeq 1 - \Phi \Bigg (\frac{T^{b}_{stat.} - \mu _{T^{b}_{stat.}}}{\sigma _{T^{b}_{stat.}}}\Bigg ),$$
  where $\Phi$ denotes the cumulative distribution function of the standard normal distribution.
7. The mid *p* value of $T^{b}_{stat.}$ at the observed value $T^{b}_{0}$ is approximated using the SPA, as defined in Eq. (16).
8. The simulated (reference) mid *p* value, which is EMPV is computed as
  $$mid\text {-}pv(T^{b}_{0}) = \frac{\sum \big (I(T^{b}_{stat.} > T^{b}_{0}) + \frac{1}{2} \sum I(T^{b}_{stat.} = T^{b}_{0})\big )}{10^5},$$
  where the summation is taken over all $10^5$ simulated values of the test statistic.
9. Steps 1 through 8 were repeated 1, 000 times to obtain replicated results.
10. The averages of the 1, 000 EMPVs, SPA *p* values, and NA *p* values were computed.
11. The average relative absolute errors of the SPA and NA *p* values were calculated with respect to the EMPVs, as defined below.
  $$\text {R.E.SPA} = \frac{1}{n_e} \sum _{j=1}^{n_e} \frac{\left| PV_{\text {SPA},j} - PV_{\text {EMPV},j}\right| }{PV_{\text {EMPV},j}}, \quad \text {R.E.NA} = \frac{1}{n_e} \sum _{j=1}^{n_e} \frac{\left| PV_{\text {NA},j} - PV_{\text {EMPV},j}\right| }{PV_{\text {EMPV},j}}$$
  where $PV_{\text {SPA},j}$, $PV_{\text {NA},j}$, and $PV_{\text {EMPV},j}$ denote the SPA, NA, and EMPVs for the *j*th replication, and $n_e = 1000$ represents the total number of replications.
12. The time required to calculate the EMPVs (T.E) and the SPA *p* values (T.S) was recorded in minutes for all replications of the proposed tests.

Tables 3 and 4 summarize the results of the simulation study. Each table reports the censoring percentage ( Cens. % $_{(if\,exist)}$), the SPA *p* value (SPA$_{p}$), the exact mid *p* value (EMPV$_{P}$), and the NA *p* value (NA$_{p}$). In addition, the evaluation measures are defined as follows: (SPA Prop.) denotes the percentage of the 1, 000 samples for which the SPA *p* value is closer to the EMPV than the NA *p* value. (R.E.SPA) and (R.E.NA) represent the average relative absolute errors of the SPA and NA *p* values, respectively. The computation times for the simulation-based method and the SPA are also reported as T.E and T.S, respectively.

**Table 1:** Simulation results under exponential and uniform distributions.

Test statistic	Sample size	Cens. % $_{(if\,exist)}$	EMPV$_{P}$	SPA$_{p}$	NA$_{p}$	SPA Prop.	R.E.SPA	R.E.NA	T.E$_{(min)}$	T.S$_{(min)}$
	$$N=15$$									
$T^{b}_{LR}$		$25\%$ Cens.	0.2182	0.2183	0.2154	0.955	0.0052	0.0242	120.1	0.2
$T^{b}_{LR}$		$50\%$ Cens.	0.2005	0.2005	0.1981	0.904	0.0115	0.0325	124.8	0.2
$T^{b}_{WLR}$			0.2013	0.2013	0.1984	0.939	0.0109	0.1075	92.9	0.2
$T^{b}_{LRC}$			0.1284	0.1284	0.1258	0.947	0.0065	0.0256	75.5	0.2
$T^{b}_{WC}$			0.0548	0.0549	0.0551	0.747	0.0597	0.1348	71.6	0.2
	$$N=20$$									
$T^{b}_{LR}$		$25\%$ Cens.	0.0922	0.0922	0.0922	0.827	0.0485	0.1301	128.7	0.2
$T^{b}_{LR}$		$50\%$ Cens.	0.0826	0.0826	0.0826	0.810	0.0387	0.1083	159.7	0.2
$T^{b}_{WLR}$			0.1859	0.1858	0.1852	0.908	0.0326	0.3680	162.1	0.4
$T^{b}_{LRC}$			0.0706	0.0705	0.0707	0.890	0.0474	0.3021	92.9	0.2
$T^{b}_{WC}$			0.0034	0.0033	0.0041	0.995	0.0805	0.4720	109.9	0.2
	$$N=40$$									
$T^{b}_{LR}$		$25\%$ Cens.	0.1001	0.1000	0.0997	0.827	0.0213	0.0978	130.3	0.2
$T^{b}_{LR}$		$50\%$ Cens.	0.1192	0.1191	0.1187	0.797	0.0192	0.0641	134.3	0.2
$T^{b}_{WLR}$			0.1720	0.1719	0.1709	0.873	0.0135	0.0933	157.5	0.3
$T^{b}_{LRC}$			0.0399	0.0398	0.0402	0.899	0.0497	0.4129	94.7	0.2
$T^{b}_{WC}$			0.0388	0.0386	0.0388	0.687	0.0669	0.1755	110.8	0.2
	$$N=60$$									
$T^{b}_{LR}$		$25\%$ Cens.	0.0564	0.0564	0.0565	0.805	0.0451	0.1663	176.7	0.3
$T^{b}_{LR}$		$50\%$ Cens.	0.0873	0.0872	0.0870	0.738	0.0377	0.0939	175.9	0.3
$T^{b}_{WLR}$			0.1580	0.1579	0.1572	0.821	0.0172	0.0631	150.8	0.5
$T^{b}_{LRC}$			0.0721	0.0720	0.0720	0.830	0.0306	0.1501	115.4	0.2
$T^{b}_{WC}$			0.0120	0.0120	0.0122	0.746	0.1292	0.2795	128.6	0.3
	$$N=80$$									
$T^{b}_{LR}$		$25\%$ Cens.	0.0367	0.0366	0.0368	0.744	0.0592	0.1505	230.5	0.4
$T^{b}_{LR}$		$50\%$ Cens.	0.0654	0.0653	0.0653	0.650	0.0369	0.0707	227.5	0.4
$T^{b}_{WLR}$			0.1355	0.1354	0.1350	0.779	0.0156	0.0493	287.5	1.3
$T^{b}_{LRC}$			0.0877	0.0876	0.0875	0.790	0.0260	0.0889	152.6	0.4
$T^{b}_{WC}$			0.0112	0.0112	0.0114	0.666	0.1385	0.2324	160.3	0.4

**Table 2:** Simulation results under extreme value and uniform distributions.

Test statistic	Sample size	Cens. % $_{(if\,exist)}$	EMPV$_{P}$	SPA$_{p}$	NA$_{p}$	SPA Prop.	R.E.SPA	R.E.NA	T.E$_{(in\,minutes)}$	T.S$_{(in\,minutes)}$
	$$N=15$$									
$T^{b}_{LR}$		$25 \%$ Cens.	0.2253	0.2253	0.2227	0.944	0.0058	0.0266	175.3	0.2
$T^{b}_{LR}$		$50\%$ Cens.	0.2114	0.2114	0.2087	0.920	0.0084	0.0290	181.8	0.2
$T^{b}_{WLR}$			0.2175	0.2175	0.2141	0.946	0.0076	0.1042	255.7	0.5
$T^{b}_{LRC}$			0.1049	0.1049	0.1029	0.880	0.0087	0.0249	103.5	0.1
$T^{b}_{WC}$			0.0494	0.0495	0.0500	0.872	0.0510	0.1406	144.2	0.3
	$$N=20$$									
$T^{b}_{LR}$		$25 \%$ Cens.	0.1218	0.1218	0.1212	0.828	0.0267	0.0750	217.6	5.1
$T^{b}_{LR}$		$50\%$ Cens.	0.1237	0.1238	0.1231	0.836	0.0249	0.0645	213.8	4.9
$T^{b}_{WLR}$			0.2212	0.2212	0.2198	0.886	0.0147	0.0994	217.0	8.1
$T^{b}_{LRC}$			0.1369	0.1367	0.1360	0.868	0.0226	0.1207	146.1	1.0
$T^{b}_{WC}$			0.0630	0.0631		0.960	0.0372	0.2286	146.6	1.1
	$$N=40$$									
$T^{b}_{LR}$		$25 \%$ Cens.	0.2151	0.2150	0.2140	0.826	0.0060	0.0185	230.9	9.4
$T^{b}_{LR}$		$50\%$ Cens.	0.2221	0.2220	0.2211	0.803	0.0056	0.0159	222.4	8.8
$T^{b}_{WLR}$			0.2247	0.2246	0.2232	0.846	0.0070	0.0314	228.3	14.7
$T^{b}_{LRC}$			0.0961	0.0959	0.0957	0.877	0.0264	0.1770	150.3	1.1
$T^{b}_{WC}$			0.1144	0.1144	0.1139	0.748	0.0175	0.0353	151.0	1.2
	$$N=60$$									
$T^{b}_{LR}$		$25 \%$ Cens.	0.2109	0.2108	0.2099	0.812	0.0060	0.0152	266.5	12.6
$T^{b}_{LR}$		$50\%$ Cens.	0.2156	0.2156	0.2149	0.741	0.0055	0.0110	266.5	12.5
$T^{b}_{WLR}$			0.2193	0.2192	0.2181	0.826	0.0060	0.0197	277.9	22.4
$T^{b}_{LRC}$			0.0331	0.0330	0.0332	0.871	0.0848	0.4071	183.6	1.3
$T^{b}_{WC}$			0.0637	0.0638	0.0638	0.684	0.0355	0.0712	180.1	1.5
	$$N=80$$									
$T^{b}_{LR}$		$25 \%$ Cens.	0.2091	0.2090	0.2085	0.731	0.0064	0.0134	355.4	14.6
$T^{b}_{LR}$		$50 \%$ Cens.	0.2027	0.2026	0.2021	0.691	0.0064	0.0116	355.3	14.7
$T^{b}_{WLR}$			0.2075	0.2074	0.2066	0.782	0.0067	0.0172	363.1	24.9
$T^{b}_{LRC}$			0.0183	0.0183	0.0186	0.799	0.1178	0.3725	306.9	1.8
$T^{b}_{WC}$			0.0169	0.0169	0.0170	0.628	0.1273	0.2168	237.5	1.8

**Table 3:** Simulation results under logistic and uniform distributions.

Test statistic	Sample size	Cens. % $_{(if\,exist)}$	EMPV$_{P}$	SPA$_{p}$	NA$_{p}$	SPA Prop.	R.E.SPA	R.E.NA	T.E$_{(min)}$	T.S$_{(min)}$
	$$N=15$$									
$T^{b}_{LR}$		$25\%$ Cens.	0.1085	0.1086	0.1062	0.892	0.0081	0.0258	229.1	0.4
$T^{b}_{LR}$		$50\%$ Cens.	0.1060	0.1060	0.1045	0.820	0.0321	0.0588	223.8	0.3
$T^{b}_{WLR}$			0.1818	0.1816	0.1809	0.584	0.0280	0.0332	182.7	0.5
$T^{b}_{LRC}$			0.2013	0.2013	0.1985	0.959	0.0071	0.0273	178.5	0.4
$T^{b}_{WC}$			0.0493	0.0494	0.0499	0.864	0.0521	0.1440	188.7	0.4
	$$N=20$$									
$T^{b}_{LR}$		$25 \%$ Cens.	0.0027	0.0028	0.0036	0.899	0.3587	0.7308	382.5	0.5
$T^{b}_{LR}$		$50\%$ Cens.	0.0082	0.0082	0.0091	0.789	0.1200	0.2679	294.4	0.4
$T^{b}_{WLR}$			0.0295	0.0295	0.0306	0.901	0.1589	0.5735	252.6	0.6
$T^{b}_{LRC}$			0.0083	0.0084	0.0096	0.966	0.1253	0.9617	236.5	0.5
$T^{b}_{WC}$			0.0033	0.0034	0.0041	0.852	0.2388	0.6606	243.5	0.6
	$$N=40$$									
$T^{b}_{LR}$		$25 \%$ Cens.	0.1631	0.1630	0.1623	0.821	0.0112	0.0444	291.1	0.4
$T^{b}_{LR}$		$50\%$ Cens.	0.1725	0.1725	0.1718	0.763	0.0110	0.0302	277.1	0.4
$T^{b}_{WLR}$			0.0075	0.0075	0.0080	0.938	0.1821	1.1043	266.0	0.6
$T^{b}_{LRC}$			0.0039	0.0039	0.0044	0.974	0.2420	2.1120	241.3	0.5
$T^{b}_{WC}$			0.1287	0.1287	0.1283	0.760	0.0479	0.0809	229.4	0.4
	$$N=60$$									
$T^{b}_{LR}$		$25 \%$ Cens.	0.0820	0.0819	0.0819	0.754	0.0258	0.0874	370.4	0.5
$T^{b}_{LR}$		$50\%$ Cens.	0.1203	0.1202	0.1120	0.736	0.0151	0.0373	354.7	0.5
$T^{b}_{WLR}$			0.0815	0.0814	0.0813	0.784	0.0459	0.1612	455.0	1.5
$T^{b}_{LRC}$			0.0010	0.0010	0.0012	0.909	0.3365	1.5679	302.6	0.8
$T^{b}_{WC}$			0.1205	0.1205	0.1203	0.660	0.0437	0.0582	337.1	0.7
	$$N=80$$									
$T^{b}_{LR}$		$25 \%$ Cens.	0.0019	0.0018	0.0020	0.763	0.3050	0.7431	704.3	1.0
$T^{b}_{LR}$		$50 \%$ Cens.	0.0092	0.0091	0.0093	0.740	0.1375	0.2894	465.3	1.0
$T^{b}_{WLR}$			0.0394	0.0394	0.0395	0.737	0.0659	0.1863	575.7	2.4
$T^{b}_{LRC}$			0.0115	0.0115	0.0019	0.840	0.1337	0.4779	403.2	1.3
$T^{b}_{WC}$			0.1319	0.1319	0.1317	0.592	0.0241	0.0293	312.4	0.7

**Table 4:** Simulation results under Weibull and uniform distributions.

Test statistic	Sample size	Cens. % $_{(if\,exist)}$	EMPV$_{P}$	SPA$_{p}$	NA$_{p}$	SPA Prop.	R.E.SPA	R.E.NA	T.E$_{(min)}$	T.S$_{(min)}$
	$$N=15$$									
$T^{b}_{LR}$		$25 \%$ Cens.	0.1474	0.1475	0.1448	0.924	0.0069	0.0239	180.0	0.2
$T^{b}_{LR}$		$50\%$ Cens.	0.1471	0.1471	0.1450	0.910	0.0169	0.0429	174.2	0.2
$T^{b}_{WLR}$			0.1257	0.1257	0.1248	0.924	0.0322	0.2540	169.0	0.3
$T^{b}_{LRC}$			0.1597	0.1596	0.1570	0.943	0.0061	0.0247	122.5	0.2
$T^{b}_{WC}$			0.0500	0.0500	0.0503	0.763	0.0611	0.1416	129.1	0.3
	$$N=20$$									
$T^{b}_{LR}$		$25 \%$ Cens.	0.0258	0.0258	0.0266	0.837	0.1140	0.2983	219.6	0.2
$T^{b}_{LR}$		$50\%$ Cens.	0.0388	0.0387	0.0393	0.783	0.0701	0.1762	224.9	0.3
$T^{b}_{WLR}$			0.0561	0.0562	0.0575	0.932	0.1124	0.5674	212.7	0.4
$T^{b}_{LRC}$			0.0113	0.0114	0.0126	0.973	0.1221	0.8925	1384.1	0.3
$T^{b}_{WC}$			0.0020	0.0020	0.0026	0.998	0.1182	0.6597	155.3	0.2
	$$N=40$$									
$T^{b}_{LR}$		$25 \%$ Cens.	0.0094	0.0093	0.0098	0.914	0.1192	0.6821	220.9	0.6
$T^{b}_{LR}$		$50\%$ Cens.	0.0257	0.056	0.0260	0.827	0.0600	0.2525	217.2	0.3
$T^{b}_{WLR}$			0.0348	0.0347	0.0352	0.893	0.0586	0.4579	220	0.4
$T^{b}_{LRC}$			0.0005	0.0005	0.0008	0.980	0.3656	3.2761	162.2	0.2
$T^{b}_{WC}$			0.0038	0.0038	0.0040	0.795	0.2460	0.8310	156.7	0.2
	$$N=60$$									
$T^{b}_{LR}$		$25\%$ Cens.	0.0579	0.0578	0.0578	0.790	0.0425	0.1498	269.9	0.4
$T^{b}_{LR}$		$50\%$ Cens.	0.0974	0.0974	0.0972	0.716	0.0213	0.0516	260.9	0.3
$T^{b}_{WLR}$			0.0135	0.0134	0.0138	0.882	0.1224	0.5384	268.8	0.6
$T^{b}_{LRC}$			0.0243	0.0243	0.0246	0.887	0.0911	0.4700	242.4	0.3
$T^{b}_{WC}$			0.0180	0.0180	0.0182	0.731	0.0984	0.2034	210.8	0.4
	$$N=80$$									
$T^{b}_{LR}$		$25 \%$ Cens.	0.0127	0.0126	0.0129	0.750	0.1308	0.3422	346.8	0.6
$T^{b}_{LR}$		$50\%$ Cens.	0.0307	0.0307	0.0308	0.688	0.0714	0.1426	525.3	0.6
$T^{b}_{WLR}$			0.0053	0.0053	0.0056	0.837	0.2018	0.6373	358.3	1.2
$T^{b}_{LRC}$			0.0276	0.0278	0.0281	0.825	0.0888	0.3072	259.2	0.6
$T^{b}_{WC}$			0.0105	0.0105	0.0107	0.672	0.1406	0.2601	281.7	0.7

**Table 5:** Simulation results under log-logistic and uniform distributions.

Test statistic	Sample size	Cens. % $_{(if\,exist)}$	EMPV$_{P}$	SPA$_{p}$	NA$_{p}$	SPA Prop.	R.E.SPA	R.E.NA	T.E $_{(in\,minutes)}$	T.S $_{(in\,minutes)}$
	$$N=15$$									
$T^{b}_{LR}$		$25 \%$ Cens.	0.2286	0.2286	0.2259	0.964	0.0070	0.0334	68.4	0.2
$T^{b}_{LR}$		$50\%$ Cens.	0.2196	0.2196	0.2169	0.920	0.0066	0.0277	64.4	0.1
$T^{b}_{WLR}$			0.2209	0.2209	0.2177	0.922	0.0087	0.0657	94.6	0.2
$T^{b}_{LRC}$			0.1890	0.1890	0.1864	0.932	0.0065	0.0260	118.4	0.2
$T^{b}_{WC}$			0.0655	0.0655	0.0655	0.778	0.0508	0.1105	77.2	0.2
	$$N=20$$									
$T^{b}_{LR}$		$25 \%$ Cens.	0.1224	0.1224	0.1219	0.847	0.0215	0.0719	211.3	2.2
$T^{b}_{LR}$		$50\%$ Cens.	0.1284	0.1284	0.1277	0.828	0.0226	0.0596	214.8	2.5
$T^{b}_{WLR}$			0.2460	0.2424	0.2389	0.850	0.0477	0.0779	214.3	3.1
$T^{b}_{LRC}$			0.1608	0.1607	0.1596	0.905	0.0151	0.0795	148.3	0.9
$T^{b}_{WC}$			0.0685	0.0686	0.0691	0.972	0.0368	0.2272	150.0	0.9
	$$N=40$$									
$T^{b}_{LR}$		$25\%$ Cens.	0.2119	0.2118	0.2107	0.839	0.0054	0.0179	218.7	3.4
$T^{b}_{LR}$		$50\%$ Cens.	0.2085	0.2084	0.2074	0.803	0.0056	0.0163	215.1	3.4
$T^{b}_{WLR}$			0.2510	0.2501	0.2481	0.798	0.0097	0.0276	215.7	5.2
$T^{b}_{LRC}$			0.2130	0.2128	0.2115	0.874	0.0060	0.0277	152.1	1.0
$T^{b}_{WC}$			0.1294	0.1294	0.1289	0.720	0.0150	0.0302	154.3	1.1
	$$N=60$$									
$T^{b}_{LR}$		$25\%$ Cens.	0.2197	0.2195	0.2187	0.789	0.0054	0.0130	421.3	5.4
$T^{b}_{LR}$		$50\%$ Cens.	0.2156	0.2155	0.2148	0.719	0.0068	0.0132	416.5	5.0
$T^{b}_{WLR}$			0.2535	0.2529	0.2516	0.715	0.0081	0.0152	263.6	5.9
$T^{b}_{LRC}$			0.2144	0.2143	0.2134	0.844	0.0058	0.0201	183.4	1.0
$T^{b}_{WC}$			0.1482	0.1483	0.1479	0.640	0.0116	0.0213	185.2	1.3
	$$N=80$$									
$T^{b}_{LR}$		$25 \%$ Cens.	0.2111	0.2110	0.2105	0.707	0.0054	0.0107	420.2	6.2
$T^{b}_{LR}$		$50 \%$ Cens.	0.2045	0.2044	0.2040	0.665	0.0065	0.0099	428.7	6.1
$T^{b}_{WLR}$			0.2499	0.2496	0.2487	0.679	0.0078	0.0127	352.2	8.1
$T^{b}_{LRC}$			0.1878	0.1878	0.1872	0.745	0.0189	0.0313	285.6	1.4
$T^{b}_{WC}$			0.1418	0.1419	0.1416	0.597	0.0108	0.0150	241.1	1.7

### Interpretation and summary of simulation findings

The simulation findings reported in Tables 1, 2, 3, 4 and 5 highlight the clear advantage of the SPA method over NA. As an illustration, Table 1 shows that when the sample size is $N = 15$, with $25\%$ censoring , and the test statistic considered is $T^{b}_{LR}$, the SPA$_{p}$ aligned more closely with the EMPV$_{p}$ in $95.5\%$ of the replications. Under these conditions, the R.E.SPA was only $0.52\%$, whereas NA produced a considerably larger R.E.NA of $2.42\%$. In a comparable manner, Table 4 reveals that, for a sample size of $N = 60$ and test statistic $T^{b}_{LRC}$, the SPA *p* values demonstrated closer correspondence with the EMPV$_{p}$ in $88.7\%$ of the replications. Under these conditions, the R.E.SPA was restricted to $9.11\%$, whereas NA exhibited a markedly higher R.E.NA of $47.0\%$. The comparison of T.S and T.E results confirms that SPA substantially reduces computation time compared to the simulation-based procedure required to obtain the EMPV$_{p}$. In summary, the SPA method achieves not only higher accuracy but also greater computational efficiency.

Considering the average performance across all simulation runs, the following results were observed: For $T^{b}_{LR}$, $25\%$ and $50\%$ censoring, as well as $T^{b}_{WLR}$, $T^{b}_{LRC}$ and $T^{b}_{WC}$, SPA *p* values were consistent with EMPV$_{p}$ in $83.2\%$, $78.4\%$, $83.9\%$, $88.9\%$ and $76.5\%$ of replications, respectively. The $T^{b}_{LRC}$ statistic recorded the greatest superiority proportion SPA Prop. among all tests.

To assess the overall approximation performance across all simulation settings, the relative errors were summed over all considered scenarios for each underlying distribution. The results showed that SPA consistently produced smaller total relative errors than NA under all distributions considered.

The smallest total relative errors were observed under the log-logistic distribution, indicating the most stable approximation performance for both methods. In contrast, the logistic and Weibull distributions produced the largest total relative errors, suggesting that these settings were more challenging for the approximation procedures.

The advantage of SPA became more noticeable under the more difficult distributions. For example, under the Weibull distribution, the total relative error of NA was approximately 4.6 times larger than that of SPA, while under the logistic distribution it was about 3.8 times larger. These findings indicate that SPA provides better numerical stability and more accurate approximation than NA, especially under distributions with more complicated tail behavior or skewness. To assess the calibration of the proposed tests under the null hypothesis, empirical Type-I error rates were computed for all four test statistics across the various sample sizes ($N = 15, 20, 40, 60, 80$), nominal significance levels ($\alpha = 0.01$ and $\alpha = 0.05$), and all considered distributional scenarios. Tables 6, 7, 8, 9 and 10 report these empirical rates across the investigated distributions.

**Table 6:** Empirical Type-I error rates under the exponential distribution for different significance levels.

Test statistic	Cens. %	$\alpha$	Sample size									
			$N=15$		$N=20$		$N=40$		$N=60$		$N=80$	
			SPA	NA	SPA	NA	SPA	NA	SPA	NA	SPA	NA
$T^{b}_{LR}$	$25\%$	0.01	0.007	0.005	0.011	0.008	0.013	0.013	0.011	0.009	0.009	0.008
		0.05	0.051	0.051	0.066	0.066	0.050	0.050	0.047	0.046	0.051	0.050
$T^{b}_{LR}$	$50\%$	0.01	0.012	0.008	0.012	0.009	0.009	0.008	0.011	0.011	0.011	0.011
		0.05	0.050	0.047	0.054	0.052	0.046	0.046	0.046	0.045	0.052	0.052
$T^{b}_{WLR}$		0.01	0.007	0.005	0.009	0.007	0.011	0.009	0.009	0.009	0.006	0.006
		0.05	0.050	0.048	0.051	0.051	0.062	0.062	0.044	0.043	0.061	0.061
$T^{b}_{LRC}$		0.01	0.009	0.007	0.009	0.009	0.011	0.008	0.008	0.005	0.008	0.006
		0.05	0.052	0.050	0.053	0.053	0.055	0.054	0.054	0.053	0.057	0.057
$T^{b}_{WC}$		0.01	0.009	0.009	0.012	0.009	0.01	0.008	0.011	0.005	0.011	0.009
		0.05	0.060	0.060	0.063	0.063	0.049	0.049	0.053	0.047	0.047	0.045

**Table 7:** Empirical Type-I error rates under the extreme value distribution for different significance levels.

Test statistic	Cens. %	$\alpha$	Sample Size									
			$N=15$		$N=20$		$N=40$		$N=60$		$N=80$	
			SPA	NA	SPA	NA	SPA	NA	SPA	NA	SPA	NA
$T^{b}_{LR}$	$25\%$	0.01	0.009	0.006	0.01	0.007	0.011	0.009	0.005	0.005	0.012	0.012
		0.05	0.041	0.040	0.059	0.059	0.053	0.053	0.051	0.051	0.055	0.055
$T^{b}_{LR}$	$50\%$	0.01	0.006	0.006	0.009	0.003	0.007	0.006	0.009	0.009	0.007	0.007
		0.05	0.055	0.057	0.050	0.046	0.051	0.051	0.050	0.050	0.050	0.050
$T^{b}_{WLR}$		0.01	0.008	0.007	0.010	0.008	0.013	0.010	0.014	0.014	0.004	0.004
		0.05	0.053	0.054	0.059	0.058	0.053	0.053	0.063	0.062	0.037	0.037
$T^{b}_{LRC}$		0.01	0.006	0.005	0.011	0.011	0.010	0.007	0.008	0.008	0.010	0.008
		0.05	0.041	0.040	0.058	0.058	0.050	0.050	0.050	0.050	0.049	0.048
$T^{b}_{WC}$		0.01	0.010	0.012	0.016	0.013	0.008	0.005	0.015	0.010	0.006	0.006
		0.05	0.063	0.065	0.067	0.067	0.035	0.035	0.048	0.040	0.0036	0.034

**Table 8:** Empirical Type-I error rates under the logistic distribution for different significance levels.

Test statistic	Cens. %	$\alpha$	Sample Size									
			$N=15$		$N=20$		$N=40$		$N=60$		$N=80$	
			SPA	NA	SPA	NA	SPA	NA	SPA	NA	SPA	NA
$T^{b}_{LR}$	$25\%$	0.01	0.011	0.007	0.010	0.008	0.009	0.007	0.009	0.008	0.008	0.008
		0.05	0.054	0.052	0.055	0.055	0.048	0.048	0.043	0.043	0.044	0.044
$T^{b}_{LR}$	$50\%$	0.01	0.009	0.007	0.008	0.004	0.008	0.007	0.006	0.005	0.006	0.006
		0.05	0.065	0.066	0.047	0.043	0.046	0.046	0.042	0.042	0.041	0.041
$T^{b}_{WLR}$		0.01	0.004	0.004	0.012	0.009	0.010	0.010	0.007	0.007	0.013	0.011
		0.05	0.050	0.048	0.050	0.049	0.059	0.059	0.047	0.047	0.041	0.041
$T^{b}_{LRC}$		0.01	0.009	0.008	0.007	0.007	0.006	0.005	0.006	0.006	0.011	0.011
		0.05	0.045	0.044	0.060	0.060	0.044	0.044	0.047	0.047	0.054	0.054
$T^{b}_{WC}$		0.01	0.008	0.008	0.016	0.013	0.009	0.006	0.014	0.003	0.009	0.007
		0.05	0.050	0.050	0.067	0.064	0.057	0.057	0.049	0.038	0.041	0.039

**Table 9:** Empirical Type-I error rates under the Weibull distribution for different significance levels.

Test statistic	Cens. %	$\alpha$	Sample Size									
			$N=15$		$N=20$		$N=40$		$N=60$		$N=80$	
			SPA	NA	SPA	NA	SPA	NA	SPA	NA	SPA	NA
$T^{b}_{LR}$	$25\%$	0.01	0.012	0.007	0.011	0.008	0.010	0.009	0.017	0.017	0.013	0.013
		0.05	0.052	0.051	0.053	0.053	0.054	0.053	0.049	0.049	0.049	0.049
$T^{b}_{LR}$	$50\%$	0.01	0.009	0.003	0.009	0.005	0.010	0.009	0.011	0.010	0.011	0.010
		0.05	0.053	0.050	0.061	0.057	0.056	0.056	0.047	0.047	0.048	0.048
$T^{b}_{WLR}$		0.01	0.010	0.004	0.013	0.005	0.010	0.008	0.008	0.007	0.009	0.009
		0.05	0.055	0.056	0.063	0.062	0.046	0.046	0.043	0.043	0.059	0.059
$T^{b}_{LRC}$		0.01	0.009	0.007	0.010	0.010	0.009	0.008	0.007	0.006	0.009	0.009
		0.05	0.054	0.052	0.063	0.063	0.048	0.048	0.054	0.054	0.055	0.055
$T^{b}_{WC}$		0.01	0.009	0.007	0.014	0.005	0.009	0.008	0.010	0.007	0.010	0.009
		0.05	0.050	0.048	0.056	0.054	0.054	0.054	0.048	0.048	0.051	0.050

**Table 10:** Empirical Type-I error rates under the log-logistic distribution across different significance levels.

Test statistic	Cens. %	$\alpha$	Sample Size									
			$N=15$		$N=20$		$N=40$		$N=60$		$N=80$	
			SPA	NA	SPA	NA	SPA	NA	SPA	NA	SPA	NA
$T^{b}_{LR}$	$25\%$	0.01	0.009	0.006	0.007	0.007	0.007	0.007	0.011	0.010	0.009	0.008
		0.05	0.049	0.048	0.041	0.041	0.053	0.053	0.054	0.054	0.050	0.050
$T^{b}_{LR}$	$50\%$	0.01	0.009	0.009	0.007	0.006	0.013	0.013	0.013	0.013	0.006	0.005
		0.05	0.054	0.057	0.047	0.047	0.051	0.051	0.055	0.055	0.052	0.052
$T^{b}_{WLR}$		0.01	0.007	0.004	0.006	0.004	0.010	0.009	0.009	0.008	0.009	0.008
		0.05	0.047	0.047	0.053	0.052	0.051	0.050	0.042	0.042	0.041	0.041
$T^{b}_{LRC}$		0.01	0.010	0.008	0.009	0.009	0.008	0.007	0.010	0.009	0.007	0.005
		0.05	0.050	0.050	0.050	0.050	0.041	0.041	0.046	0.046	0.051	0.051
$T^{b}_{WC}$		0.01	0.011	0.008	0.016	0.013	0.016	0.011	0.013	0.008	0.012	0.009
		0.05	0.048	0.049	0.067	0.064	0.055	0.055	0.050	0.042	0.059	0.056

Across all evaluated configurations, both the SPA and NA empirical Type-I error rates remain well-controlled and close to their nominal thresholds. Specifically, at $\alpha = 0.05$, the simulated rates generally fall within the [0.04, 0.07] interval, while at $\alpha = 0.01$, they lie within [0.006, 0.017]. These bounds confirm stable size control, exhibiting no systematic inflation or deflation as the sample size or underlying distribution varies.

**Fig. 1:**
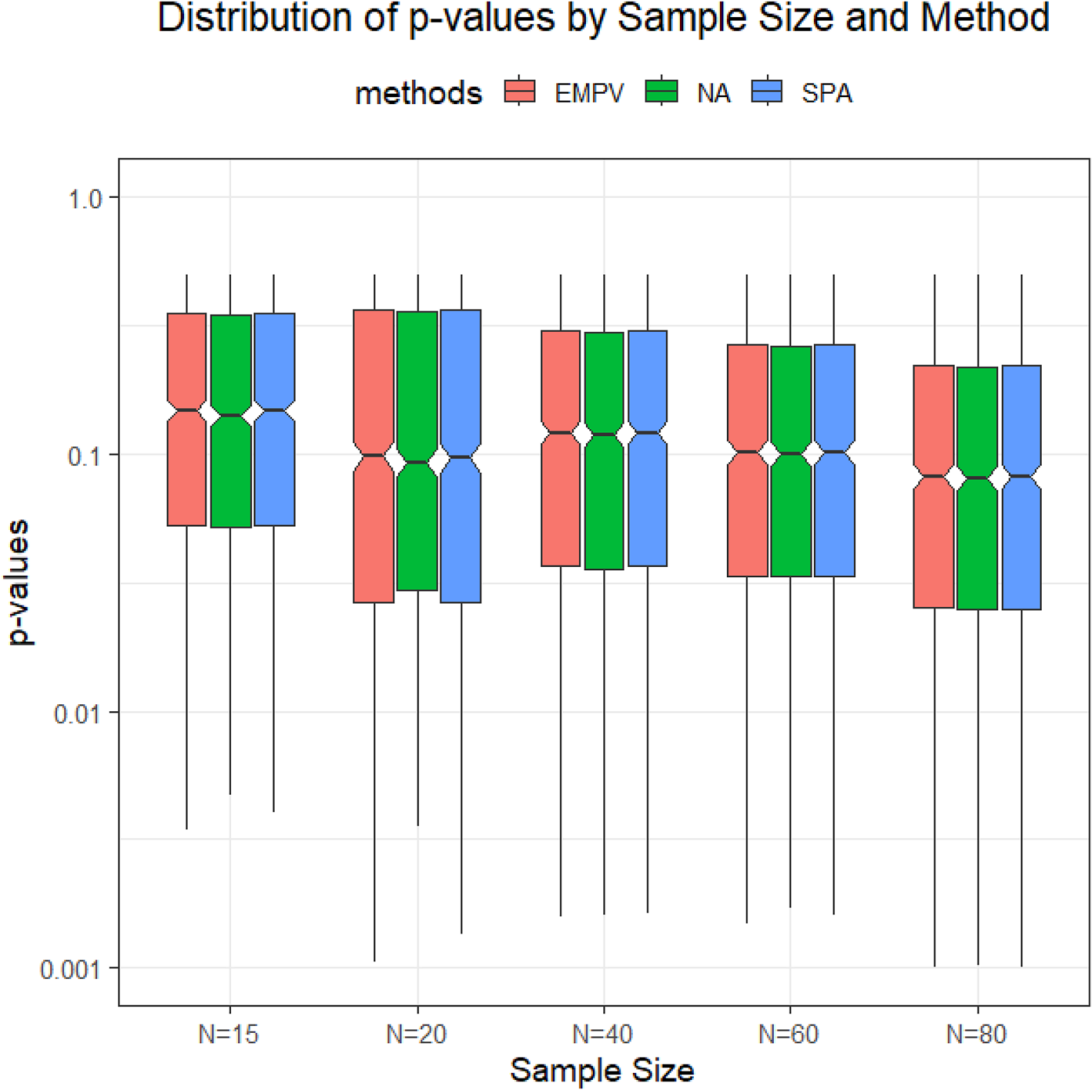
EMPV, NA, and SPA *p* values for $T^{b}_{WLR}$ under exponential distribution.

**Fig. 2:**
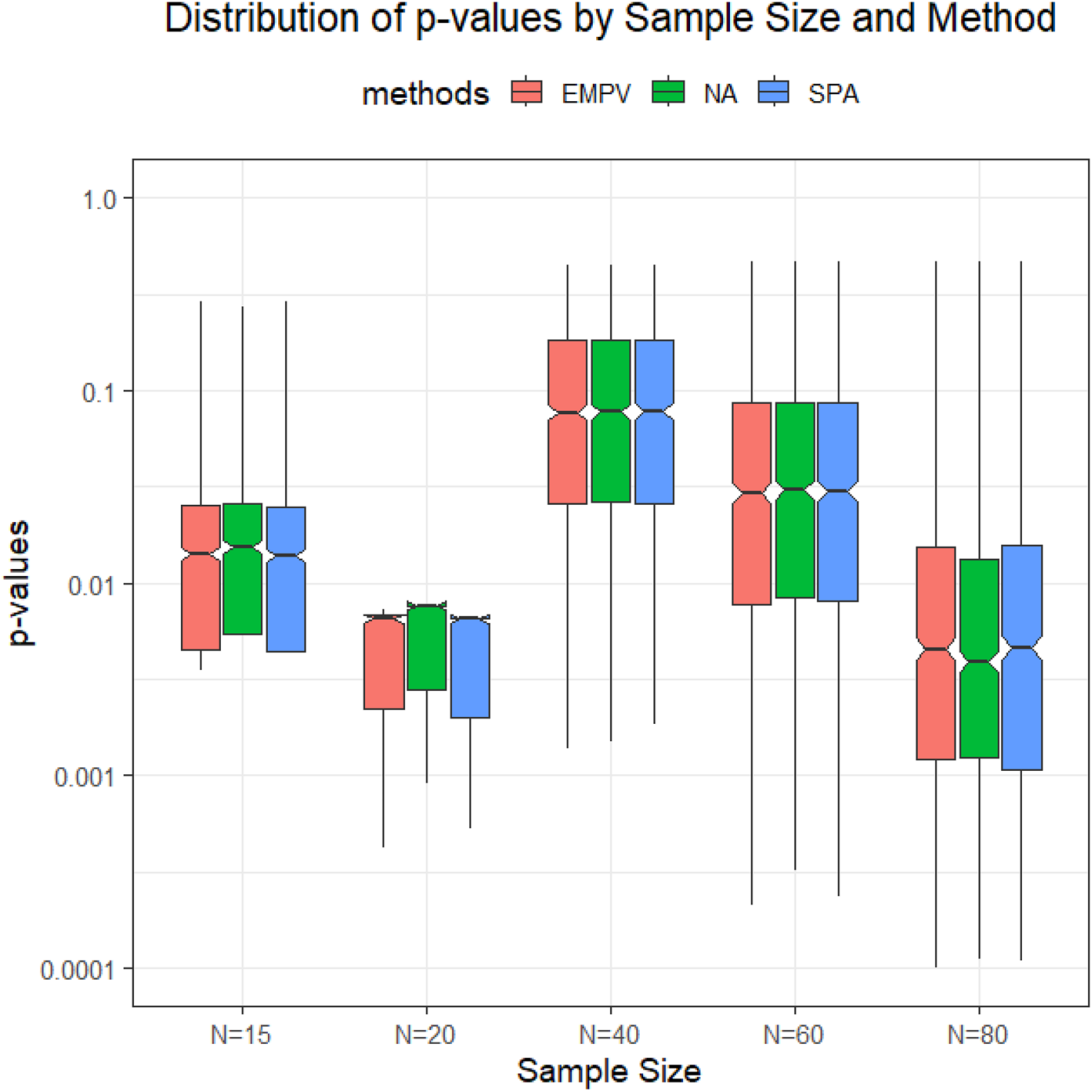
EMPV, NA, and SPA *p* values for $T^{b}_{WC}$ under extreme value distribution.

**Fig. 3:**
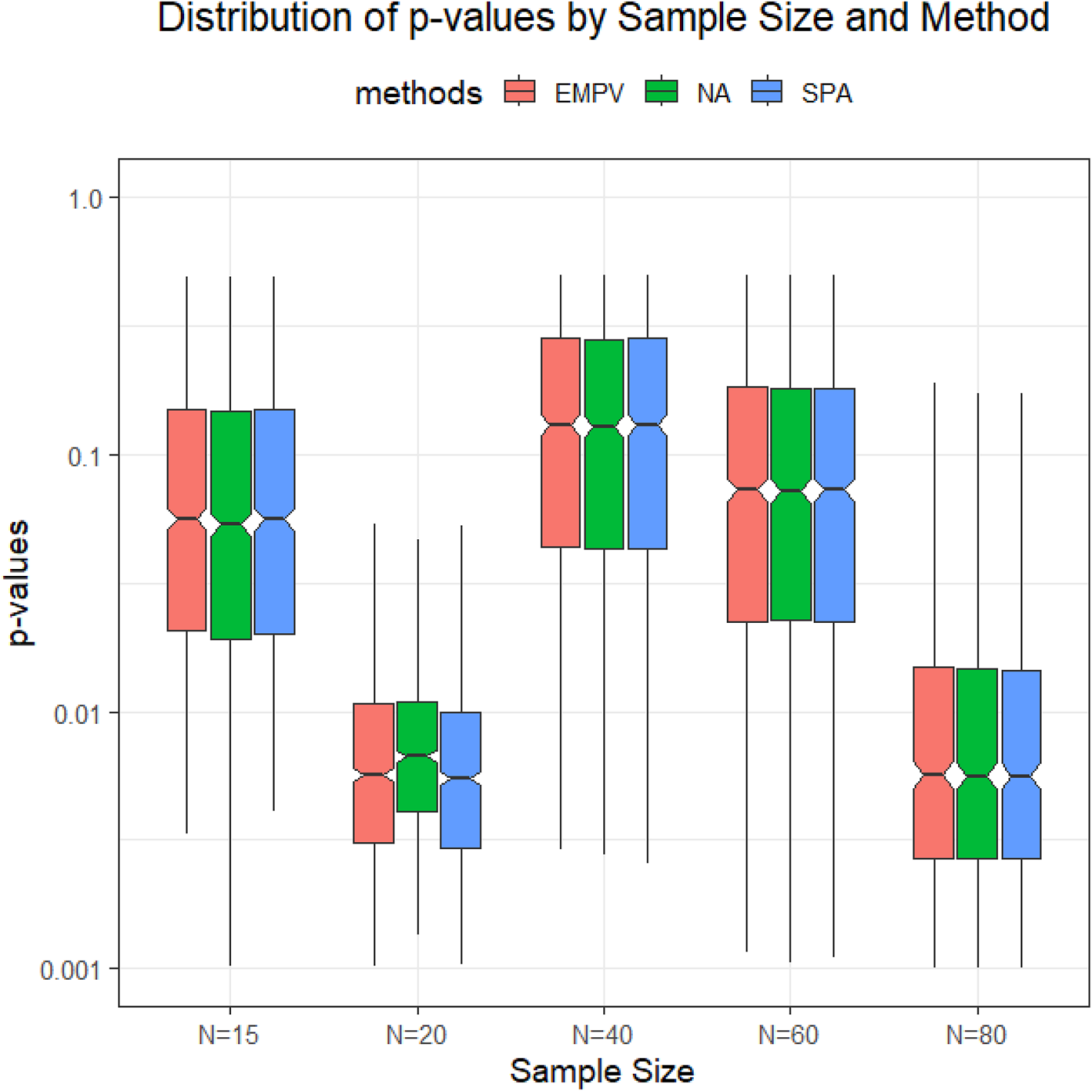
EMPV, NA, and SPA *p* values for $T^{b}_{LR}$ ($50\%$ Cens.) under logistic distribution.

**Fig. 4:**
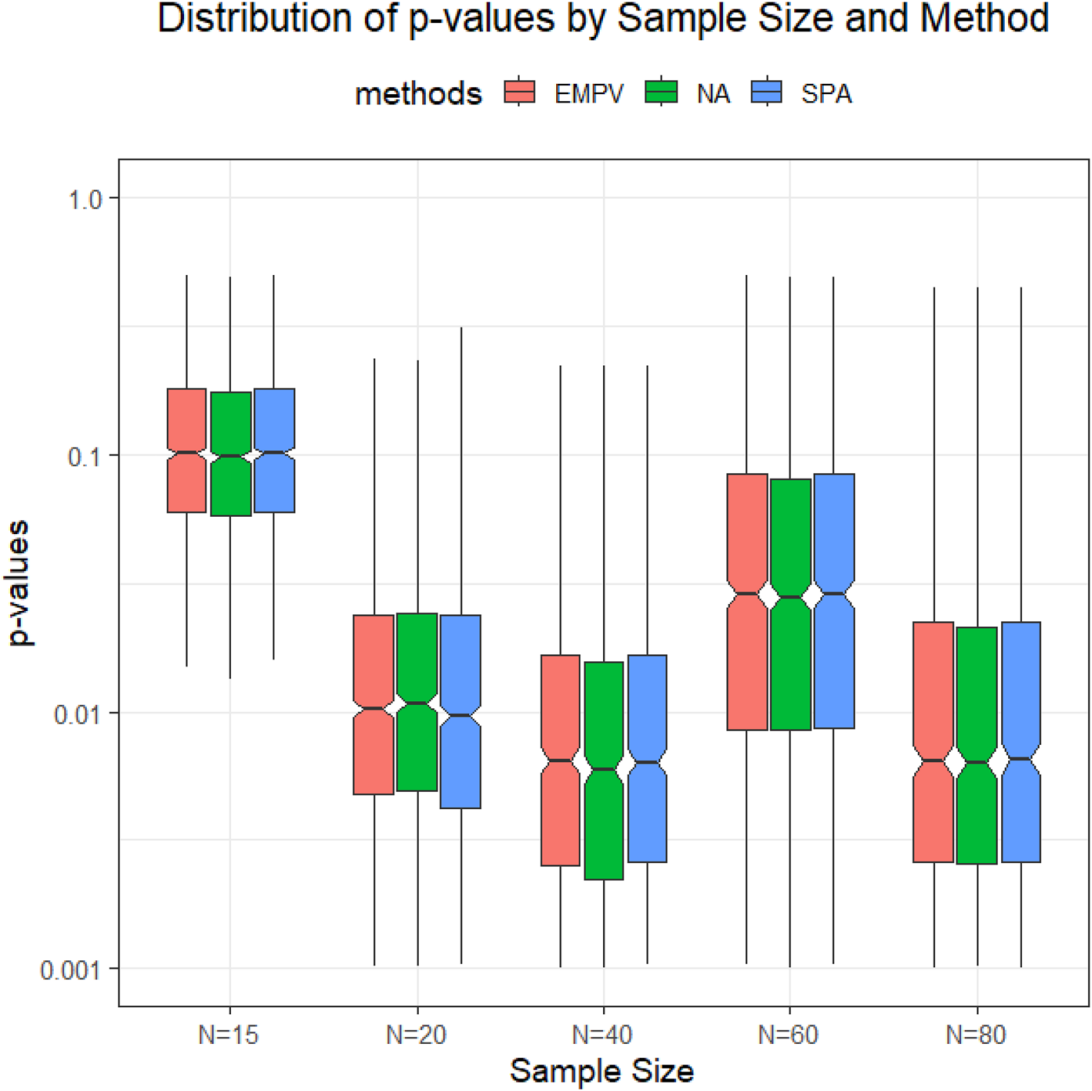
EMPV, NA, and SPA *p* values for $T^{b}_{LR}$ ($25\%$ Cens.) under weibull distribution.

**Fig. 5:**
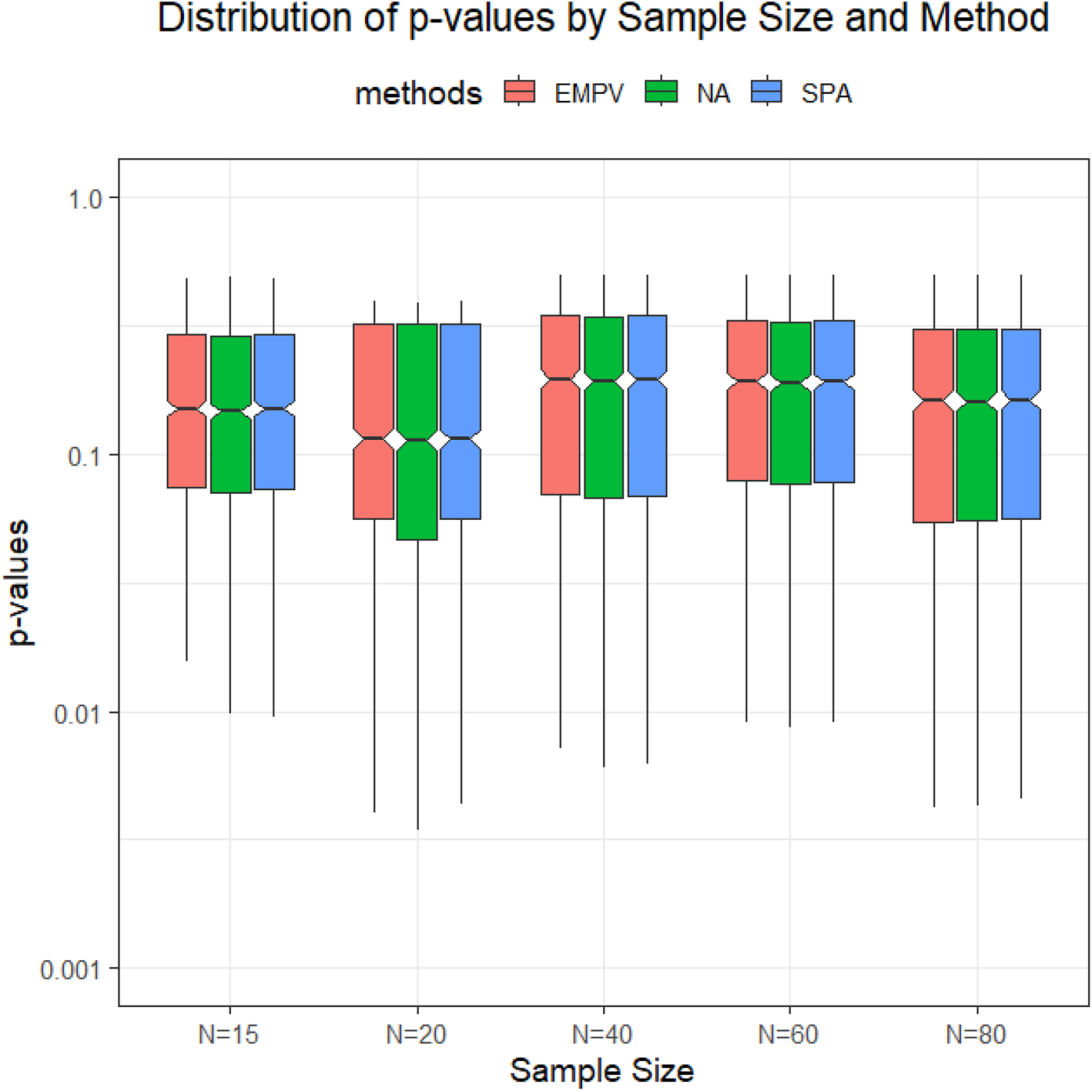
EMPV, NA, and SPA *p* values for $T^{b}_{LRC}$ under log-logistic distribution.

Crucially, while Type-I error tables verify that both methods successfully bound the false-positive rate at a fixed binary threshold ($p < \alpha$), they do not reflect the overall structural accuracy of the approximation across the entire distribution. The essential advantage of the SPA over the NA is captured instead by the *p*-value empirical boxplots (Figs. 1, 2, 3, 4 and 5) and relative error tables. Under small sample sizes ($N \le 20$), heavy-tailed profiles Fig. 1, and highly skewed data (Fig. 2), the asymptotic symmetry assumption of the NA breaks down, causing its distribution to deviate markedly from the exact empirical baseline EMPV. Conversely, the SPA accurately fits the underlying asymmetry and matches the exact EMPV distribution across all support points. Thus, the Type-I error rates demonstrate that both tests are valid, but the distributional analysis confirms that the SPA achieves this validity by tracking the true shape of the test statistic rather than relying on asymptotic convergence.

Figures 1, 2, 3, 4 and 5 contain box plots that compare the *p* values produced by the SPA and NA methods for the proposed tests across various sample sizes. They were used to illustrate how the *p* values distributed and median changes for each method across the different test statistics. For instance, as illustrated in Fig. 2 for sample size 80, the SPA box plot exhibit higher median than the NA box plot. This suggests that the SPA approach generally yields larger *p* values. The observed pattern at $N=20$ may be attributed to a more favorable balance between sampling variability and statistical power. At this sample size, the test appears to achieve greater stability, resulting in smaller and more homogeneous *p* values compared with neighboring sample sizes. Consequently, the inter quartile range (IQR) is reduced and the corresponding boxplot appears more compact. This behavior does not necessarily indicate any issue with the data; rather, it may reflect the intrinsic characteristics of the sampling process and the statistical properties of the test at this particular sample size. Furthermore, Fig. 4 shows that for $N=60$ and $N=80$, all three methods (EXACT, NA, and SPA) display tight distributions with medians slightly below 0.05, while for sample size $N=40$, the SPA box plot exhibit higher median than the NA box plot.

**Fig. 6:**
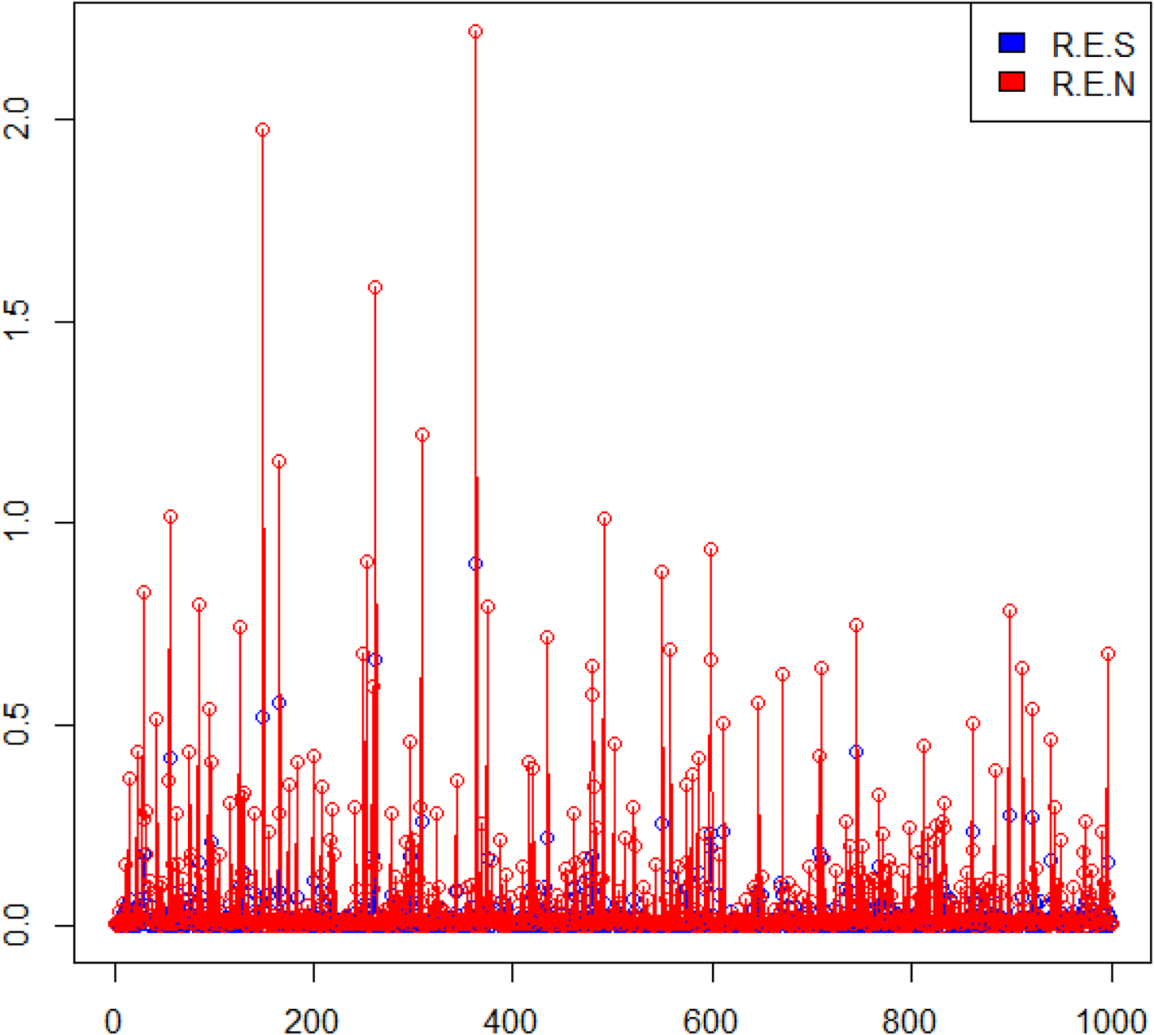
Error comparison for $T^{b}_{LR}$ from log-logistic distribution: SPA vs. NA ($N=20$, 25% censoring).

**Fig. 7:**
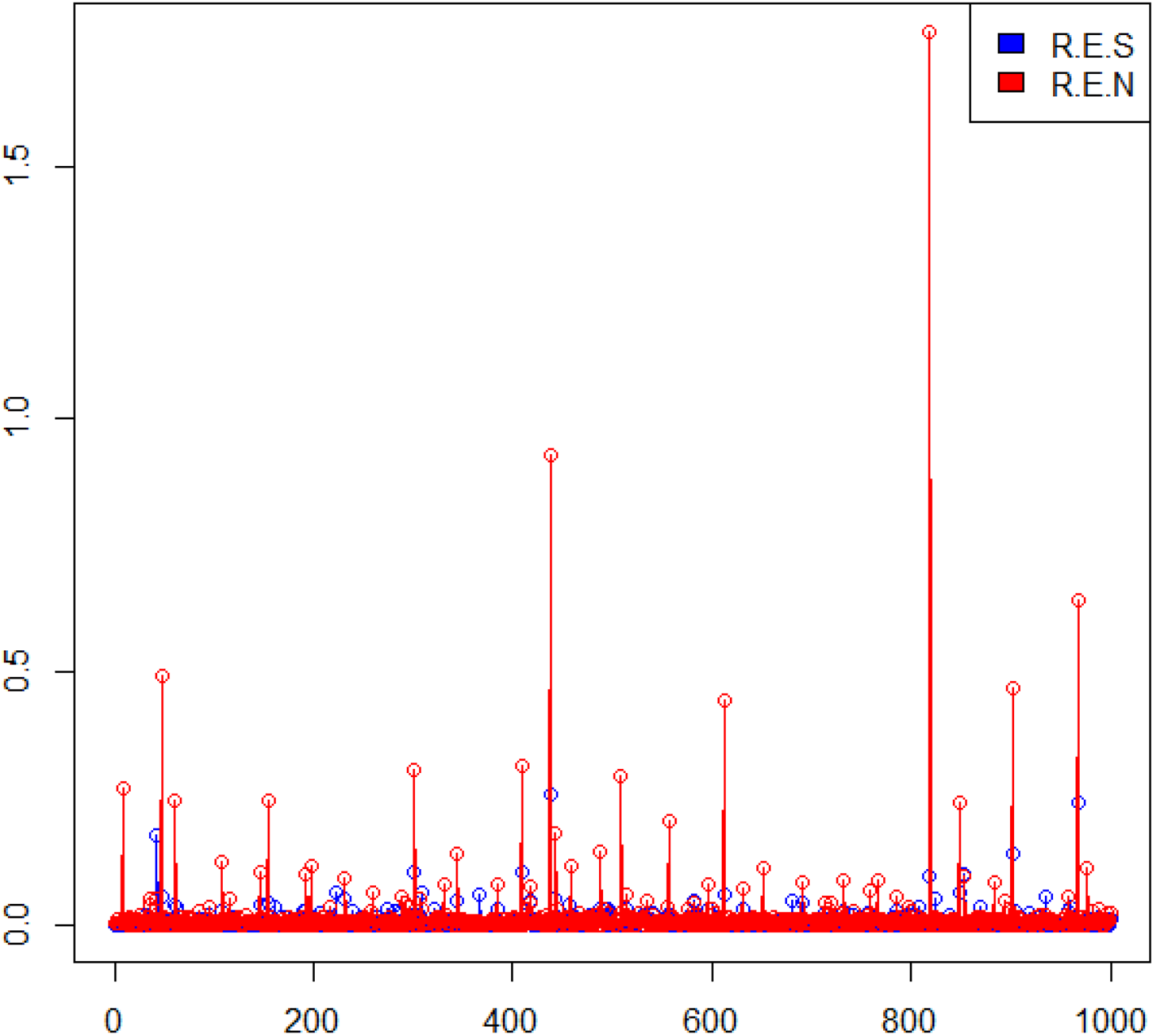
Error comparison for $T^{b}_{WLR}$ from extreme value distribution: SPA vs. NA ($N=80$).

**Fig. 8:**
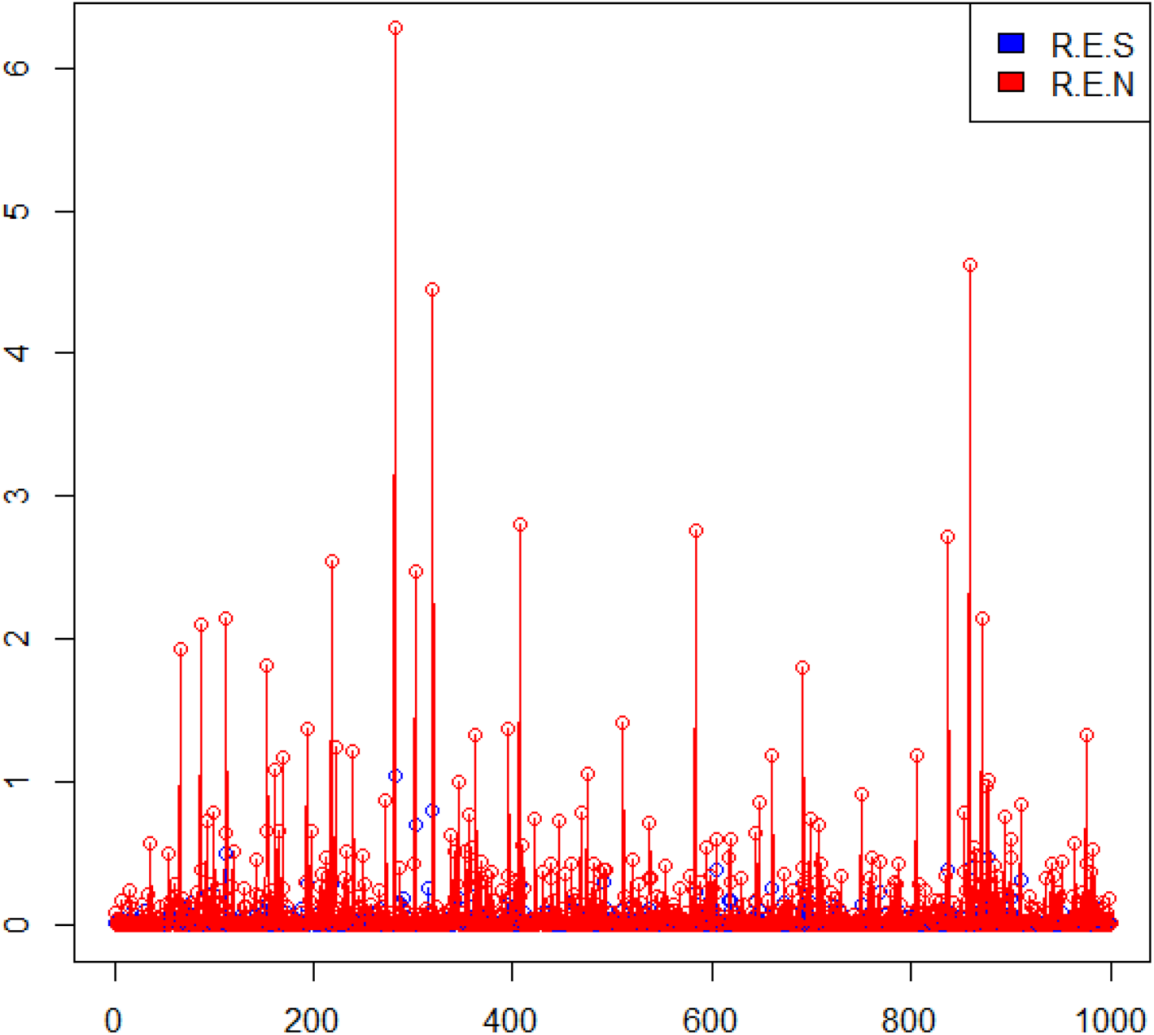
Error comparison for $T^{b}_{LRC}$ from exponential distribution: SPA vs. NA ($N=60$).

**Fig. 9:**
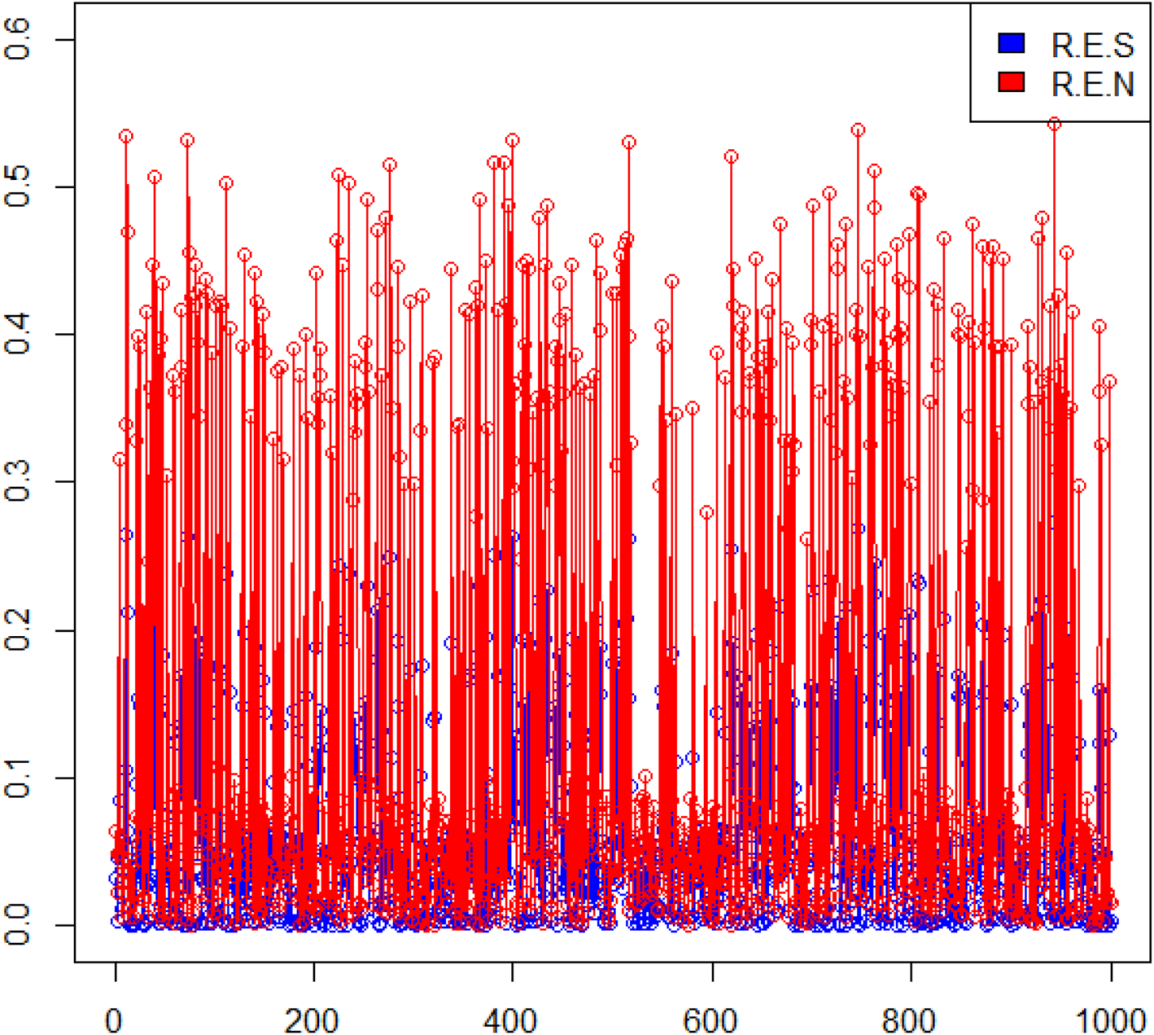
Error comparison for $T^{b}_{WC}$ from weibull distribution: SPA vs. NA ($N=15$).

Figures 6, 7, 8 and 9 provide additional visual comparisons based on some considered cases, illustrating that the NA consistently shows greater error rates than the SPA across all tested statistics.

Figures 6, 7, 8 and 9 compare the R.E.SPA and R.E.NA over 1, 000 simulation runs. The results demonstrate the clear and consistent superiority of the SPA; while the NA errors fluctuate wildly with extreme, unstable spikes due to its failure under skewed or heavy-tailed distributions, the SPA errors remain uniformly low and tightly bound near zero across all replications. Even in scenarios where the NA breaks down severely, the corresponding SPA errors remain completely negligible. These sharp contrasts confirm that the SPA ensures significantly better calibration and precision by maintaining strict point-wise accuracy and entirely eliminating the large approximation failures inherent in standard asymptotic methods.

## Data case studies

Patients were selected from the target population via simple random sampling. To account for left truncation, only individuals whose event times exceeded their truncation times were retained, irrespective of whether their survival times were censored or fully observed. The sample was then partitioned into five clinics, each serving as a block. Within the *i*-th block, a random allocation scheme assigned $m_{i}$ patients to the treatment group (*T*) and the remaining $n_{i} - m_{i}$ to the control group (*C*), ensuring that all $$\left( {\begin{array}{c}n_{i}\\ m_{i}\end{array}}\right)$$ possible assignment combinations were equally probable. The precise definitions of the treatment and control groups are provided within each respective case study.

Five distinct real-world datasets were used to assess the reliability of the SPA method in approximating mid *p* values for the proposed test statistics. The first dataset under consideration is the Psychiatric (Psy.) Patients dataset. It was initially introduced by Klein and Moeschberger^3^ as a subset of a more extensive investigation into inpatient psychiatric cases that Tsuang and Woolson^34^ originally documented. Their detailed analysis covered mental health patients admitted to hospitals affiliated with the University of Iowa between 1935 and 1948. Each record includes four key attributes: the patient’s age at first hospital admission, sex, the duration of follow-up (from admission until death or censoring), and the final vital status. The Psy. Patients dataset is used to illustrate the accuracy of the SPA method in approximating EMPVs for the test statistic $T^{b}_{LR}$ defined in Eq. 3, which is specifically designed for left-truncated and right-censored data. For this demonstration, the Psy. Patients dataset was utilized, with female patients designated as group *T* and male patients designated as group *C*. We constructed a sample of $N=20$ patients was partitioned into 5 blocks of 4 patients each. Within every block, the experimental treatments were allocated equally among the comparison groups.

The second dataset is the AIDS dataset originally reported by Lagakos et al.^10^. The records detail infection and illness onset for 258 adults and 37 children who had contracted the AIDS virus and developed symptoms by June 30, 1986. Data variables differ by group: adult records contain the time from April 1, 1978, to the estimated infection via contaminated blood transfusion, as well as the length of time from infection to the appearance of AIDs symptoms. In contrast, since pediatric infection commonly occurs perinatally, the infection time for children is measured as the duration from April 1, 1978, until delivery. This dataset is subsequently used to gauge the performance of the SPA method in approximating the EMPVs for test statistics $T^{b}_{WLR}$, $T^{b}_{LRC}$, and $T^{b}_{WC}$ as given in Eqs. 6, 8 and 10, respectively. The analysis confirms that the SPA methodology provides reliable approximations for data that is characterized as right-truncated and cross-sectional. We used the AIDS dataset, defining children as group *T* and adults as group *C*. Two random samples were constructed: the first consisted of 16 patients divided into 4 blocks of size 4, while the second consisted of 30 patients divided into 5 blocks of size 6.

The third dataset is the Worcester Heart Attack Study (WHAS) documented by Hosmer et al.^5^. This long-term study, conducted biennially from 1975 to 2001, aimed to assess whether females survived longer than males after a myocardial infarction, MI. The cohort consists of patients hospitalized for acute MI, where the primary event of interest is post-hospitalization death from any cause. Key variables include the patient’s age at hospitalization and their age at the point of death or censoring. This WHAS data allows us to determine the SPA method’s reliability in calculating the approximate EMPVs for the test statistic $T^{b}_{LR}$ defined in Eq. 3, a measure designed for use with left-truncated and right-censored survival data. In the WHAS data, females were group *T* and males were group *C*. Two random samples were generated for the blocked design: one with $N=16$ patients organized into 4 blocks, 4 patients per block, and another with $N=30$ patients organized into 5 blocks, 6 patients per block.

The fourth dataset analyzed is the Channing House dataset, Hyde^35^. This resource contains survival information for 365 females and 97 males residing in the Channing House retirement community. Data was gathered from 1964 to 1975, during which time 176 residents died, 130 females and 46 males. For every resident, the age upon entry and the age at death or censoring were recorded. The observational nature of the setup necessitates accounting for left truncation, due to the entry requirement, and right censoring, due to study end or loss. We leverage this widely accessible dataset, available in R packages such as “asaur” and “boot”, to validate the SPA method’s precision when approximating the EMPVs for the test statistic $T^{b}_{LR}$ as defined in Eq. 3, which is specifically designed for left-truncated and right-censored survival data. Using the Channing House data, we set females as group *T* and males as group *C*. Two random samples were constructed with equal treatment balance per block: a $N=16$ resident sample, 4 blocks of 4, and a $N=30$ resident sample, 5 blocks of 6.

Finally, the Veteran’s Administration Lung Cancer Trial dataset (Lung Cancer) documented by Kalbfleisch and Prentice^36^, serves as the case study for illustrating the SPA method’s application in approximating the EMPVs for the test statistic $T^{b}_{LR}$ as presented in Eq. 3. This dataset, available in the R survival package, comprises 137 male patients with advanced, inoperable lung cancer who were randomly assigned to receive either the standard treatment or the test treatment. Key patient variables were calculated: the age at entry (in days) and the age at death or censoring (obtained by summing the survival time and entry age). For our analysis, the age of entry serves as the left truncation time, while the age at death or last follow-up functions as the event time. The event indicator was simplified and recoded as 1 for death and 0 for censoring. From the Lung Cancer data, we set the test treatment arm as group *T* and the standard treatment arm as group *C*, note: all patients in this dataset are male. For the randomized block design, two random samples were used: $N=16$ patients were divided into 4 blocks of 4 patients, and $N=30$ patients were split into 5 blocks of 6 patients. Treatments were balanced within every block.

The five datasets analyzed are Psy. Patients, AIDS, WHAS, Channing House, and Lung Cancer. Table 11 presents the EMPVs, SPA, and NA *p* values for $T^{b}_{LR}$, in Eq. 3, across the four survival datasets. Table 12 provides analogous *p* value comparisons for the statistics $T^{b}_{WLR}$, $T^{b}_{LRC}$, and $T^{b}_{WC}$ as defined by Eqs. 6, 8 and 10, respectively, using the AIDS dataset, which contains right-truncated and cross-sectional data. Both Tables 11 and 12 present the computation times, in seconds, with (T.E) referring to the time needed to compute the EMPV and (T.S) representing the time required for the SPA *p* value calculation.

**Table 11:** Comparison of *p* values and timing for the test statistic $T^{b}_{LR}$ on selected datasets.

Dataset	Sample	EMPV	SPA	NA	T.E$_{(in\,seconds)}$	T.S$_{(in\,seconds)}$
Psy. Patients	Sample 1: $N=20$	0.0920	0.0910	0.0895	10.041	0.438
WHAS	Sample 1: $N=16$	0.1911	0.1892	0.1831	8.176	0.412
	Sample 2: $N=30$	0.0371	0.0370	0.0377	10.498	0.452
Channing House	Sample 1: $N=16$	0.2350	0.2307	0.2255	8.233	0.434
	Sample 2: $N=30$	0.3078	0.3047	0.3021	10.245	0.477
Lung Cancer	Sample 1: $N=16$	0.3011	0.3021	0.2981	8.134	0.415
	Sample 2: $N=30$	0.2015	0.1982	0.1953	10.351	0.444

**Table 12:** Comparison of *p* values and timing for the statistics $T^{b}_{WLR}$, $T^{b}_{LRC}$ and $T^{b}_{WC}$ on the AIDs dataset.

Sample Size	Statistic	EMPV	SPA	NA	T.E$_{(in\,seconds)}$	T.S$_{(in\,seconds)}$
$N=16$	$T^{b}_{WLR}$	0.1167	0.1125	0.1101	8.312	0.425
	$T^{b}_{LRC}$	0.0162	0.0168	0.0185	5.339	0.337
	$T^{b}_{WC}$	0.0206	0.0254	0.0264	5.381	0.332
$N=30$	$T^{b}_{WLR}$	0.1194	0.1162	0.1135	10.187	0.474
	$T^{b}_{LRC}$	0.1012	0.0994	0.0980	6.641	0.349
	$T^{b}_{WC}$	0.1503	0.1431	0.1415	6.749	0.374

The test statistic $T^{b}_{LR}$ is inapplicable to the AIDS dataset, as the dataset contains fully observed, uncensored, event times. The EMPV is computed by generating $10^{5}$ randomized block design sequences for the *T* and *C* labels, while holding the number of recurrent events constant. This mid *p* value is defined as the proportion of times the randomized test statistic $T^{b}_{stat}$ is greater than the observed test statistic $T^{b}_{0}$, plus one-half of the proportion of times they are equal $T^{b}_{stat} = T^{b}_{0}$.

The computations are summarized in Tables 1 and 2. The results consistently indicate that the SPA method provide a closer approximation to the EMPVs than the NA method, a pattern that persists across all datasets and sample configurations presented in both tables. The reported computation times T.E and T.S, for EMPVs and SPA *p* values, respectively, confirm the superior computational efficiency of the SPA method.

To check the stability of our findings, we considered how alternative blocking configurations might affect the analysis. Even though the numerical mid-*p*-values vary slightly under different random blocking arrangements, the core inferential conclusions remain unchanged. In all cases, the developed SPA consistently provides much higher accuracy than the standard normal approximation while requiring very little computation time. This confirms that our proposed procedure is robust and reliable.

## Concluding remarks and future works

In this work, we put the SPA as a strong competitor to the NA and simulation-based approaches for approximating mid *p* values in linear rank tests. We apply the SPA framework to a variety of challenging data structures, including the notable cases of left-truncated, right-censored data and cross-sectional data without follow-up, all within the randomized block design. Across all scenarios, SPA consistently delivers greater accuracy than NA and requires far less computational effort than the simulation process. Its performance remains stable whether the data arise from real studies or are generated through simulation.

Looking ahead, we intend to broaden the use of SPA to linear rank tests conducted under alternative randomization schemes. These extensions will consider designs such as the Wei’s urn procedure, and the truncated binomial design. We also plan to explore the suitability of SPA for more complex testing situations, including those involving interval-censored data. Furthermore, extending the current two-sample framework to settings with multiple covariates represents an important direction for future research. Another promising direction concerns settings with partially observed covariates, which are common in clinical practice when some patient-level predictors are missing or incompletely recorded. Extending the proposed saddlepoint approximation framework to accommodate such settings, following the approach of Das and Basu ^37^ for feature selection in Cox models with partially observed covariates^37^, represents a natural and practically relevant avenue for further development. Expanding the method in these directions will enhance its general usefulness, improve precision, and increase its relevance to a wider range of practical applications.

## Data Availability

The datasets analyzed during the current study are available from the corresponding author on reasonable request. The simulated datasets generated during the study were produced using RStudio; the parameters for generating these data are available within the paper.
